# Comprehensive analysis of the coding and non-coding RNA transcriptome expression profiles of hippocampus tissue in tx-J animal model of Wilson's disease

**DOI:** 10.1038/s41598-023-36503-8

**Published:** 2023-06-07

**Authors:** Dan Wang, Daojun Xie, Juan Zhang, Biao Cai, Bo Yang, Lei Zhou, Xiaofeng Huang

**Affiliations:** 1 Encephalopathy Center, The First Affiliated Hospital of Anhui University of Chinese Medicine, No. 117 Meishan Road, Shushan District, Hefei, 230031 People’s Republic of China; 2 College of Integrated Chinese and Western Medicine, Anhui University of Chinese Medicine, No. 1 Qianjiang Road, Xinzhan District, Hefei, 230012 People’s Republic of China

**Keywords:** Gene regulatory networks, Genome informatics, Genome, Neurodevelopmental disorders, Cognitive neuroscience, Learning and memory

## Abstract

Wilson's disease (WD) is an autosomal recessive disorder with a genetic basis. The predominant non-motor symptom of WD is cognitive dysfunction, although the specific genetic regulatory mechanism remains unclear. Tx-J mice, with an 82% sequence homology of the ATP7B gene to the human gene, are considered the most suitable model for WD. This study employs deep sequencing to investigate the differences in RNA transcript profiles, both coding and non-coding, as well as the functional characteristics of the regulatory network involved in WD cognitive impairment. The cognitive function of tx-J mice was evaluated using the Water Maze Test (WMT). Long non-coding RNA (lncRNA), circular RNA (circRNA), and messenger RNA (mRNA) profiles were analyzed in the hippocampal tissue of tx-J mice to identify differentially expressed RNAs (DE-RNAs). Subsequently, the DE-RNAs were used to construct protein–protein interaction (PPI) networks, as well as DE-circRNAs and lncRNAs-associated competing endogenous RNA (ceRNA) expression networks, and coding-noncoding co-expression (CNC) networks. To elucidate their biological functions and pathways, the PPI and ceRNA networks were subjected to Gene Ontology (GO) and Kyoto Encyclopedia of Genes and Genomes (KEGG) pathway analysis. A total of 361 differentially expressed mRNAs (DE-mRNAs), comprising 193 up-regulated and 168 down-regulated mRNAs, 2627 differentially expressed long non-coding RNAs (DE-lncRNAs), consisting of 1270 up-regulated and 1357 down-regulated lncRNAs, and 99 differentially expressed circular RNAs (DE-circRNAs), consisting of 68 up-regulated and 31 down-regulated circRNAs, were observed in the tx-J mice group when compared to the control mice group. Gene Ontology (GO) and pathway analyses revealed that DE-mRNAs were enriched in cellular processes, calcium signaling pathways, and mRNA surveillance pathways. In contrast, the DE-circRNAs-associated competing endogenous RNA (ceRNA) network was enriched for covalent chromatin modification, histone modification, and axon guidance, whereas the DE-lncRNAs-associated ceRNA network was enriched for dendritic spine, regulation of cell morphogenesis involved in differentiation, and mRNA surveillance pathway. The study presented the expression profiles of lncRNA, circRNA, and mRNA in the hippocampal tissue of tx-J mice. Furthermore, the study constructed PPI, ceRNA, and CNC expression networks. The findings are significant in comprehending the function of regulatory genes in WD associated with cognitive impairment. These results also offer valuable information for the diagnosis and treatment of WD.

## Introduction

Wilson's disease (WD) is an autosomal recessive disorder caused by mutations in the ATP7B bile duct copper transporter, causing excessive accumulation of copper (Cu) in tissues, which was introduced by Kinnear Wilson in 1912^1^. The ATP7B gene is a P-type ATPase that can maintain proper copper concentration in the body. Mutations of this gene lead to the accumulation of copper in various tissues and organs, including the liver, brain, cornea, and kidney^2^. Thus, the clinical presentation of WD varies from an asymptomatic state to hepatic, neurological, ophthalmic, and psychiatric^3^. Liver disease can present as the first clinical manifestation of Wilson's disease (WD), with a range of symptoms that include asymptomatic elevated liver enzymes, acute hepatitis, severe hepatitis, compensated/decompensated cirrhosis, and even acute liver failure^4^. Following hepatic manifestations, neurological symptoms are the most common clinical symptom, which can present as varying degrees of movement disorders such as tremor, dystonia, or parkinsonism, and non-motor dysfunctions such as dysarthria, dysphagia, psychiatric disorders, cognitive dysfunction, personality disorders, etc.^5^. Ocular pathological copper deposition can also result in ophthalmological manifestations typical of WD, such as Kayser-Fleischer rings and sunflower cataracts^6^. In addition, copper deposition in the heart can cause cardiac arrhythmias, cardiomyopathy, etc.; kidney disease can result in tubular dysfunction, kidney stones, hypercalciuria, etc.; the skeletal system can manifest as osteoporosis, cartilage calcium, osteoarthritis, etc.; and the female reproductive system can present with irregular menstruation, infertility, habitual miscarriage, etc.^7^. Cognitive dysfunction is the main non-motor symptom in neurological systems. Kirk et al. found that cognitive impairment was present in more than half of the stable WD patients regardless of phenotype and persisted in the long-term course of the disease^8^. As one of the few treatable congenital genetic diseases, early diagnosis and timely treatment of WD could facilitate effective control^9^.

In our study, tx-J mice were used as the animal model. According to research conducted by Jackson Laboratory, copper accumulation in the hippocampus caused behavioral disturbance and memory impairment in tx-J mice^10^. Previous studies have also confirmed that over-activation of mitochondrial autophagy in tx-J mice hippocampal neurons can lead to cognitive dysfunction^11^. Furthermore, a different study reported that 82% of humans and 91% of tx-J mice share the same amino acid sequence of the WND protein, which has functional domains that contain copper-transporting ATPases^12^. Therefore, tx-J mice are a valid model for studying WD.

Non-coding RNA is a hot element in the current neuroscience field. They lack protein-coding functions, while they can regulate transcription and translation through chromatin structure^13^, RNA/protein scaffolding, sponge and epigenetic modifications, selective splicing, etc.^14^. The diversity and complexity of non-coding RNAs and related gene control networks play a vital role in regulating physiological pathologies^15^. A study exploring the gene expression profiles of hippocampus aging found that immunoglobulin dysregulation may be a potential mechanism of hippocampal aging, requiring attention to the role of hippocampal-focused immunity in the aging process^16^. Whole transcriptome sequencing and refined analysis can help identify and study ncRNAs' functions. In comparison, studies of ncRNA-miRNA-mRNA ce-network of whole transcriptome profiles in the hippocampus tissue of tx-J mice are not reported currently.

This study provides a description of the coding and non-coding RNA expression profiles in the hippocampus tissue of individuals with Wilson's disease (WD). We have identified the differentially expressed lncRNAs, circRNAs, and mRNAs, and constructed a novel protein–protein interaction (PPI) network and co-expression network. Furthermore, we employed DE-circRNAs, lncRNAs-associated ceRNA networks, and GO/KEGG pathway enrichment analysis to comprehensively predict their functions. Finally, we used quantitative real-time PCR (qRT-PCR) to confirm the expression of genes associated with WD. Our findings contribute to a better understanding of the underlying molecular mechanisms of WD and strategies for diagnosing and treating WD-related cognitive impairment.

## Results

### Behavioral testing

**Figure 1:**
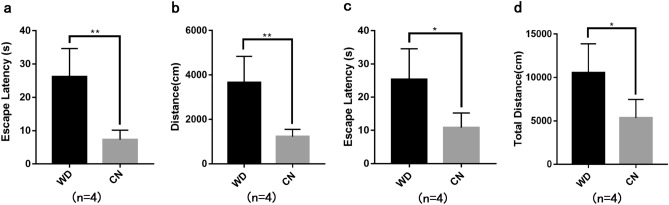
Analysis of spatial learning and memory abilities performed via MWM tests. (**a**) Escape latency of the opposite platform quadrant (**P < 0.01). (**b**) Swimming distance of the opposite platform quadrant (**P < 0.01). (**c**) Average escape latency of three quadrants except for the platform quadrant (*P < 0.05). (**d**) Total swimming distance except for the platform quadrant (*P < 0.05).

After undergoing a four-day training program, the mice were subjected to a positioning cruise experiment on the fifth day. The experiment involved comparing the escape latency and swimming distance in the quadrant opposite to the platform between the WD group and the control group. The findings indicated that the WD group had significantly higher values than the control group, with a statistically significant difference (Fig. 1a,b). Additionally, the average escape latency and total swimming distance in the three quadrants, except for the platform quadrant, were compared between the two groups. The results showed that the WD group had higher values than the control group, with a statistically significant difference (Fig. 1c,d). These results suggested that, under the same training conditions, tx-J mice require more time and swim longer distances to find the platform, indicating significantly lower spatial learning and memory abilities than normal mice.

### Hippocampus morphology

**Figure 2:**
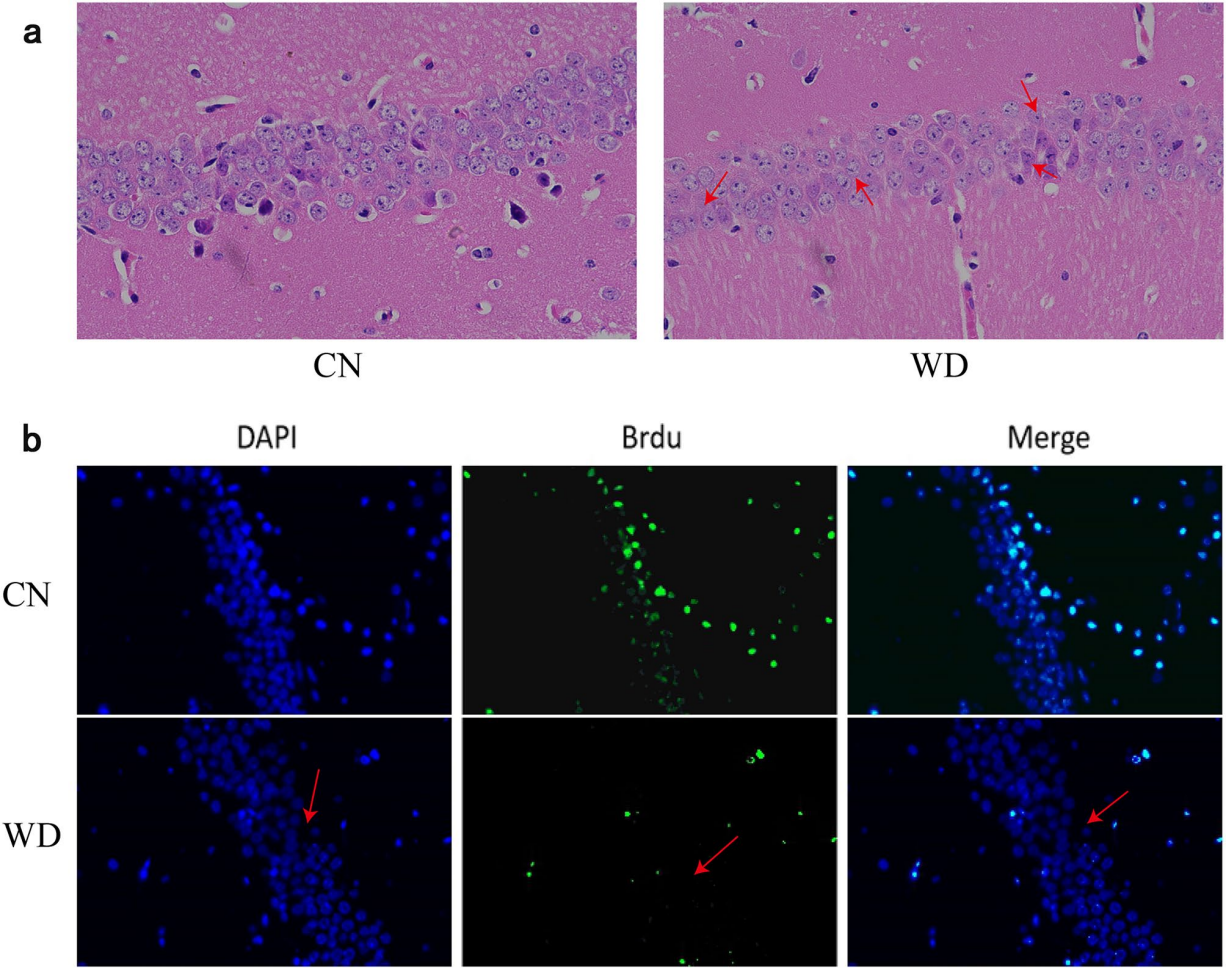
The morphological changes in the hippocampus of two groups. (**a**) The results of hematoxylin and eosin staining (× 400). The arrow in the figure shows that the hippocampal pyramidal cells are edematous, the cell gap is widened, and there is a transparent ring-like band around them, and the cell death. (**b**) The results of DAPI staining, BrdU labeling, and Merge of hippocampal neurons (× 20 μm). The arrow shows a significant decrease in the number of newly formed neurons in the hippocampus of the WD group.

As shown in Fig. 2, Haematoxylin and eosin staining results showed that the hippocampus in CA1 area pyramidal cells of control mice was densely arranged and regular, with smooth pyramidal cell nuclei, and dense peripheral nerve felt, interspersed with some glial cells, good morphology, and abundant capillaries. In contrast, the hippocampus pyramidal cells of tx-J mice in the WD group were reduced in number, loosely arranged, with widened cell intervals, heavily reduced pyramidal cell levels, mild edema, lightly stained cytoplasm and nuclei, localized pyramidal cell loss, transparent ring-like bands around the cells, and prominent cell death characteristics (Fig. 2a). The results of immunofluorescence staining showed that compared with the control group, the number of surviving new neurons in the dentate gyrus of the hippocampus in the WD group was significantly reduced (Fig. 2b).

### Genome-wide identification of RNAs in mice hippocampus

**Figure 3:**
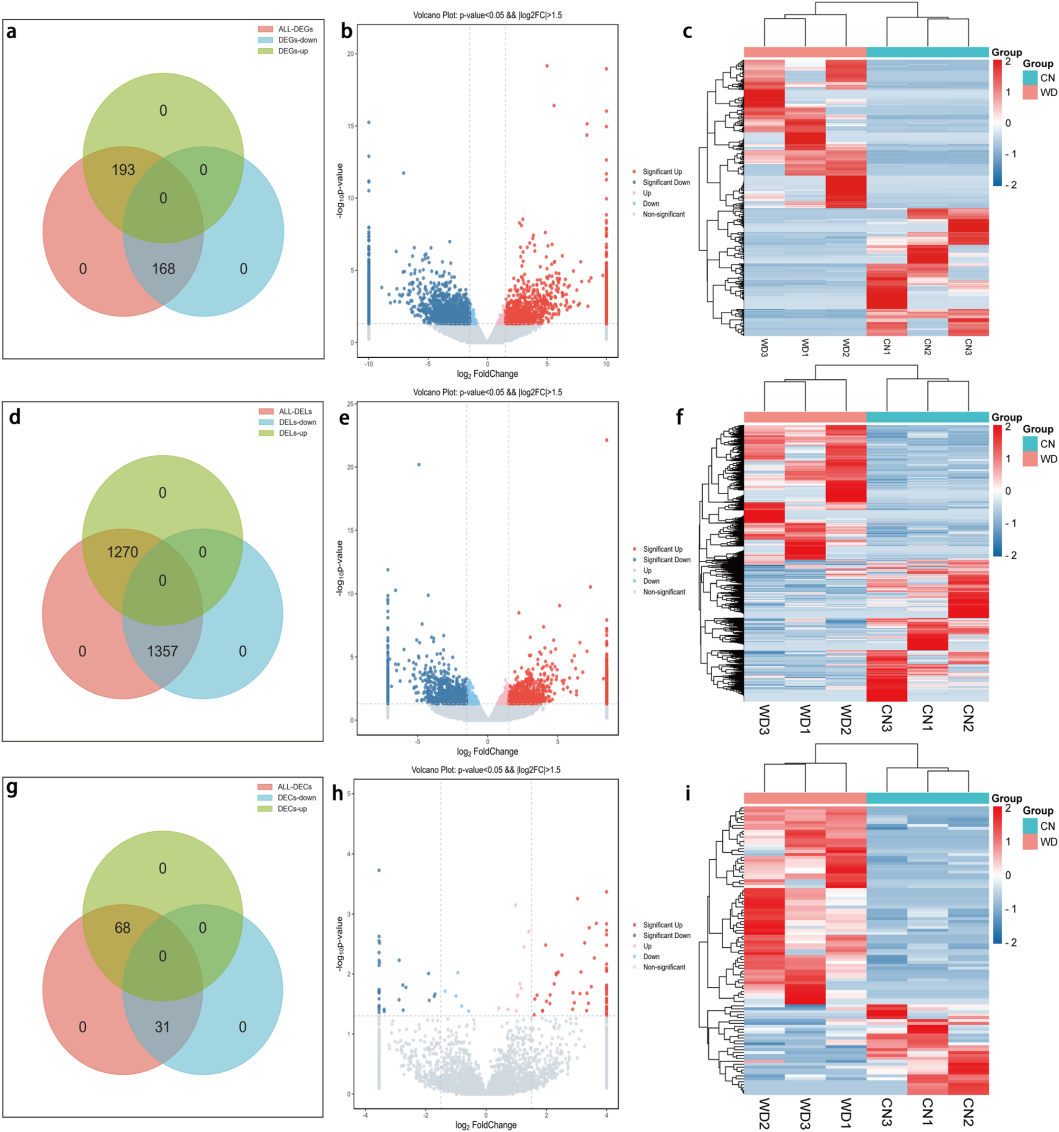
Expression profiles of DE RNAs. (**a**–**c**) Expression profiles of DE mRNAs. (**d**–**f**) Expression profiles of DE lncRNAs. (**g**–**i**) Expression profiles of DE circRNAs. The Venn diagram (**a**,**d**,**g**), which is plotted on Jvenn (http://jvenn.toulouse.inra.fr/app/example.html), shows the number of overlapping DE RNAs in the WD group compared with the control group. In volcano plots (**b**,**e**,**h**) performed using the OECloud tools (https://cloud.oebiotech.com), blue points represent down-regulated DE RNAs, while red points represent up-regulated DE RNAs. In heatmap (**c**,**f**,**i**) made with Heatmapper (http://www.heatmapper.ca/), the blue rectangles represent decreased DE RNAs, and the red rectangles represent increased DE RNAs.

We analyzed DE RNAs using the EdgeR method, following the criteria *P* < 0.05 and |log2FC|> 1.5. Venn diagram, volcano plot, and clustering map were used to show the DE RNAs, as shown in Fig. 3. Figure 3a–c indicate the Venn diagram, volcano plot, and clustering heatmap of DEGs. Figure 3d–f show the Venn diagram, volcano plot, and clustering heatmap of DELs. Figure 3g–i indicate the Venn diagram, volcano plot, and clustering heatmap of DECs. Detailed information shows in Supplementary Tables S1, S2, S3. Detailed information on the top 20 up-regulated and down-regulated DECs/DELs/DEGs in the tx-J hippocampus is demonstrated in Tables 1, 2 and 3.

**Table 1:** Detailed information of the top 10 up-regulated and down-regulated DEGs in tx-J hippocampus.

Gene id	Gene name	Log2FC	*P*-value	Up/down
ENSMUSG00000046008	Pnlip	4.083236072	0.002887232	Up
ENSMUSG00000026532	Spta1	4.038239348	6.07E−13	Up
ENSMUSG00000073400	Trim10	3.918556446	0.011296991	Up
ENSMUSG00000023216	Epb42	3.905108272	0.013568715	Up
ENSMUSG00000096002	Vmn2r53	3.712381949	0.035700177	Up
ENSMUSG00000025983	Ccdc150	3.452919949	0.021979002	Up
ENSMUSG00000114432	Gm49391	3.349967727	0.004311266	Up
ENSMUSG00000004630	Pcp2	3.188825905	0.019273485	Up
ENSMUSG00000052469	Tcp10c	3.171847496	0.006398263	Up
ENSMUSG00000026009	Icos	3.125346431	0.00279748	Up
ENSMUSG00000034486	Gbx2	− 6.323041516	0.000829043	Down
ENSMUSG00000055228	Zfp935	− 5.68536959	0.002275737	Down
ENSMUSG00000096764	Gm21985	− 5.364567547	9.41E−05	Down
ENSMUSG00000031293	Rs1	− 5.126279589	0.001526399	Down
ENSMUSG00000021919	Chat	− 5.075459088	0.013375077	Down
ENSMUSG00000027833	Shox2	− 5.001396906	0.000985249	Down
ENSMUSG00000046242	Nme9	− 4.96697126	8.91E−06	Down
ENSMUSG00000001504	Irx2	− 4.793992718	0.002018444	Down
ENSMUSG00000051980	Casr	− 4.704817667	0.002456825	Down
ENSMUSG00000061048	Cdh3	− 4.701341887	0.003674688	Down
ENSMUSG00000050377	Il31ra	− 4.694723646	7.36E−06	Down

**Table 2:** Detailed information of the top 10 up-regulated and down-regulated DECs in tx-J hippocampus.

CircRNA_id	Known_circRNA	Gene_id	Log2FC	*P*-value	Up/down
circRNA.5194	–	ENSMUSG00000015087	3.144187	0.009288	UP
circRNA.9026	–	ENSMUSG00000039716	3.26715	0.003045	UP
circRNA.1268	mmu_circ_0000374	ENSMUSG00000020955	3.331906	0.040821	UP
circRNA.5062	–	ENSMUSG00000053141	3.343849	0.020953	UP
circRNA.2061	–	ENSMUSG00000040640	3.39316	0.030786	UP
circRNA.7054	–	ENSMUSG00000010721	3.419378	0.001707	UP
circRNA.4591	–	ENSMUSG00000038349	3.488782	0.016307	UP
circRNA.2481	–	ENSMUSG00000097039	3.590303	0.005429	UP
circRNA.2225	mmu_circ_0000551	ENSMUSG00000022100	3.655628	0.001443	UP
circRNA.9007	–	ENSMUSG00000032570	3.994061	0.001886	UP
circRNA.2597	–	ENSMUSG00000016541	− 3.54879	0.00441	DOWN
circRNA.6733	–	ENSMUSG00000050017	− 3.53504	0.006462	DOWN
circRNA.2210	–	ENSMUSG00000059456	− 3.53224	0.003042	DOWN
circRNA.4168	mmu_circ_0000086	ENSMUSG00000006005	− 3.38018	0.039029	DOWN
circRNA.3396	–	ENSMUSG00000024293	− 3.37595	0.041803	DOWN
circRNA.6058	–	ENSMUSG00000028098	− 2.88716	0.027	DOWN
circRNA.218	–	ENSMUSG00000056073	− 2.8813	0.005933	DOWN
circRNA.1161	–	ENSMUSG00000035933	− 2.7606	0.015331	DOWN
circRNA.455	mmu_circ_0000331	ENSMUSG00000018412	− 2.7497	0.040005	DOWN
circRNA.742	mmu_circ_0000268	ENSMUSG00000000538	− 2.65656	0.016778	DOWN

**Table 3:** Detailed information of the top 10 up-regulated and down-regulated DELs in tx-J hippocampus.

LncRNA_id	Locus	Length	tx-J	NC	Log2FC	*P-*value	Up/Down
NONMMUT033142.2	18:77,255,711–77,262,101	6316	1.913165851	0.011822923	7.338231348	2.84E−11	UP
NONMMUT030751.2	17:80,394,058–80,396,599	2542	7.584006619	0.215910031	5.134458049	8.50E−10	UP
NONMMUT057141.2	6:57,506,497–57,508,247	1751	18.82410807	4.015538877	2.228915996	3.17E−09	UP
NONMMUT061625.2	7:59,667,720–59,686,523	1508	6.181388836	0.389138373	3.989575861	4.11E−08	UP
NONMMUT066887.2	8:101,755,393–101,763,563	4308	0.78879109	0.096087287	3.037225783	2.78E−07	UP
NONMMUT067204.2	8:110,975,325–110,975,718	394	4.937387948	0.189177721	4.705933909	4.73E−07	UP
NONMMUT001137.2	1:60,480,155–60,480,842	688	33.19687674	0.34734109	6.578550613	7.30E−07	UP
NONMMUT027930.2	16:98,063,877–98,082,001	3150	0.738731588	0.043508147	4.085692775	1.92E−06	UP
NONMMUT001322.2	1:66,719,257–66,720,829	1573	1.403992963	0.04887562	4.844276884	2.38E−06	UP
NONMMUT053361.2	5:101,832,955–101,834,962	2008	4.035558	0.02975469	7.08350728	3.59E−06	UP
NONMMUT063019.2	7:102,061,371–102,065,137	3767	0.055076214	1.656317115	− 4.910405718	6.52E−21	DOWN
NONMMUT014082.2	12:44,598,994–44,600,031	1038	0.206633626	19.70869952	− 6.575613726	5.37E−11	DOWN
ENSMUST00000142815	4:116,589,733–116,597,630	2436	0.047592957	0.901454472	− 4.243434646	1.32E−10	DOWN
NONMMUT089515.1	13:36,581,548–36,583,947	2400	0.016324739	0.419180764	− 4.682440719	2.49E−08	DOWN
NONMMUT014958.2	12:83,159,423–83,162,055	2633	0.173032432	1.525644322	− 3.140304282	2.04E−07	DOWN
NONMMUT045752.2	3:151,543,445–151,545,080	1636	0.145284886	2.332792454	− 4.005101426	2.77E−07	DOWN
NONMMUT036688.2	2:32,230,795–32,232,820	2026	0.250363815	34.96017232	− 7.12554242	3.11E−07	DOWN
NONMMUT080564.1	10:83,403,850–83,405,334	1485	0.168745401	2.437765395	− 3.852639203	3.35E−07	DOWN
NONMMUT055789.2	6:13,834,457–13,835,234	778	0.068919907	2.303726974	− 5.06290519	4.61E−07	DOWN
NONMMUT000680.2	1:39,551,303–39,555,539	1275	0.150085058	2.184852152	− 3.863683394	6.08E−07	DOWN

Overall, compared to the control group, a total of 361 DE-mRNAs (193 up-regulation and 168 down-regulation), 2627 DE-lncRNAs (1270 up-regulation and 1357 down-regulation), and 99 DE-circRNAs (68 up-regulation and 31 down-regulation) were identified in the tx-J hippocampus.

### Validation of the DECs using qRT-PCR

**Figure 4:**
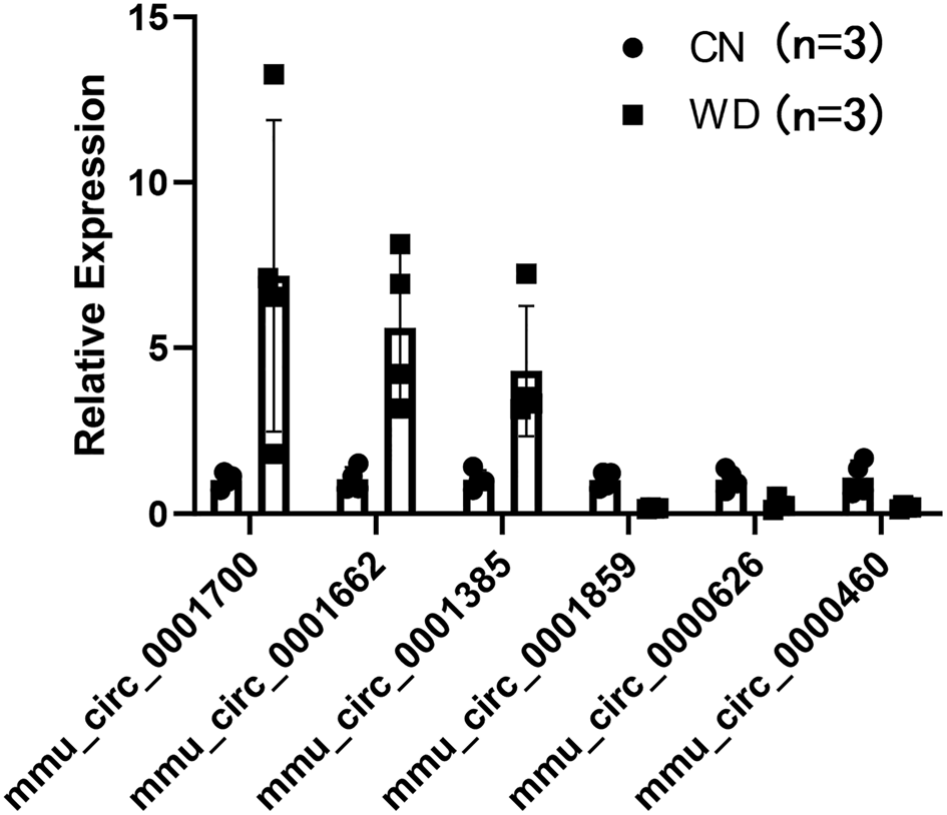
qRT-PCR validation of the DE-circRNA candidates.

Since ninety-nine circRNAs were found to have significant changes with WD in RNA-seq, the reliability of the results is unknown. To confirm the reliability, six circRNAs with relatively high-level fold changes in RNA-seq were randomly selected for qRT-PCR validation, including three up-regulated circRNAs (mmu_circ_0001700, mmu_circ_0001662, and mmu_circ_00013859) and three down-regulated circRNAs (mmu_circ_0001859, mmu_circ_0000626, and mmu_circ_0000460). As presented in Fig. 4, the qRT-PCR data show that the circRNAs mmu_circ_0001700 and mmu_circ_0001662 were significantly up-regulated, while the circRNA mmu_circ_0001859, mmu_circ_0000626, and mmu_circ_0000460 were significantly down-regulated in the WD group. These results were consistent with RNA-seq, suggesting that RNA-seq data are available.

### Construction and analysis of PPI network

**Figure 5:**
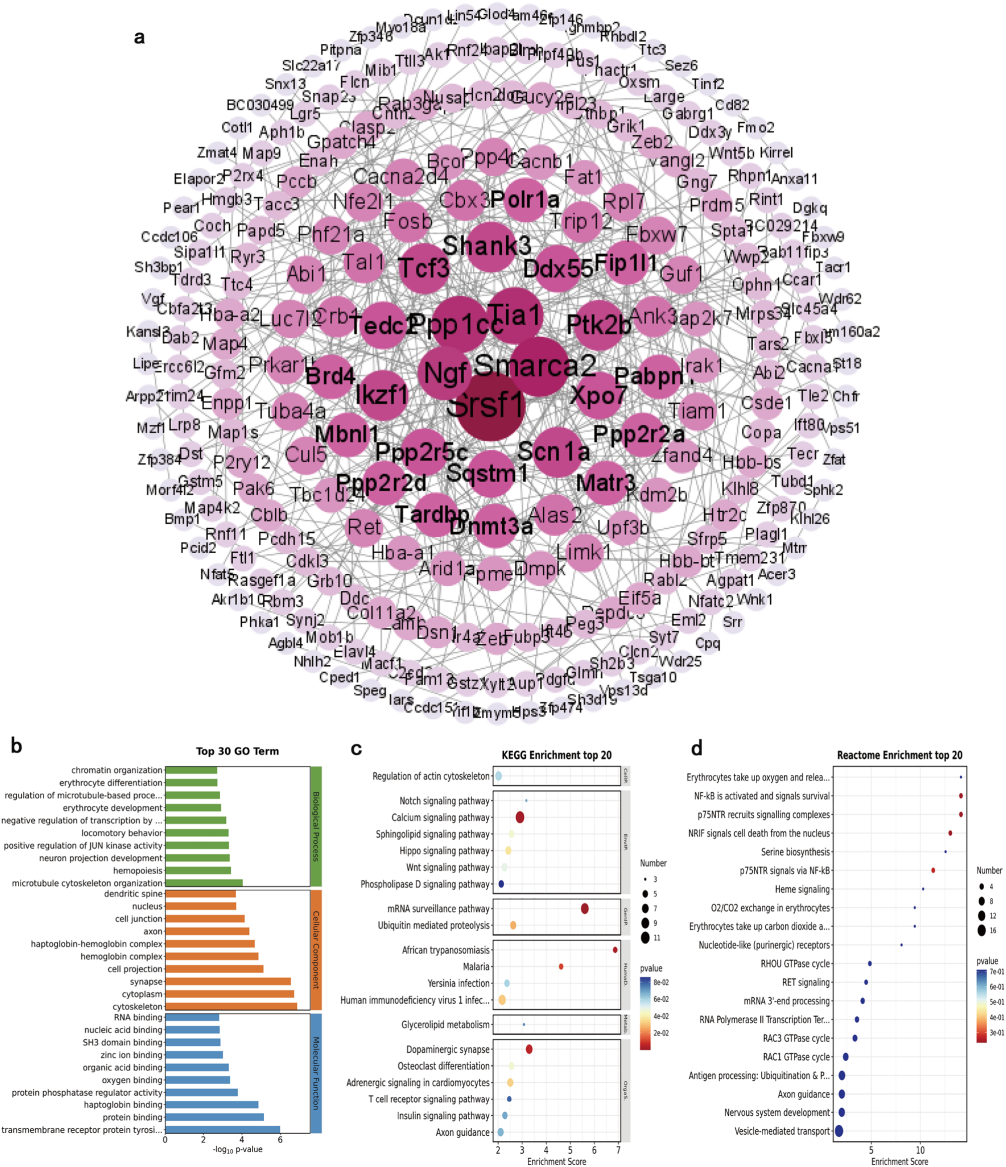
Visualizations of PPI network and functional enrichment analysis of DEGs. (**a**) PPI network was identified using Cytoscape (v3.9.1). The higher the degree value, the darker the color. (**b**) GO enrichment analyses of DEGs were performed using the OECloud tools (https://cloud.oebiotech.cn). (**c**) KEGG enrichment analyses of DEGs were performed using the OECloud tools. (**d**) Reactome enrichment analyses of DEGs were performed using the OECloud tools.

We constructed a PPI network based on the interaction of DE mRNAs with proteins shown in Fig. 5a. Detailed information on interactions is listed in Supplementary Table S4. We summarized the top ten pivotal genes with the highest degree values, which are Srsf1, Smarca2, Ppp1cc, Tia1, Ngf, Shank3, Ptk2b, Tcf3, Scn1a, and Ikzf.

GO analyses enriched in biological processes including microtubule cytoskeleton organization (GO: 0000226), hemopoiesis (GO: 0030097), and neuron projection development (GO: 0031175), the cellular component including cytoskeleton (GO: 0,005,856), cytoplasm (GO: 0005737), and synapse (GO: 0045202), molecular function including transmembrane receptor protein tyrosine kinase activator activity (GO: 0030297), protein binding (GO: 0005515), and haptoglobin binding (GO: 0031720) (Fig. 5b). KEGG pathways enriched in mRNA surveillance pathway (mmu03015), Calcium signaling pathway (mmu04020), African trypanosomiasis (mmu05143), and Dopaminergic synapse (mmu04728) et al. (Fig. 5c). Reactome enrichment was shown in Fig. 5d, such as p75NTR recruits signaling complexes (R-MMU-209543), NF-kB is activated and signals survival (R-MMU-209560), and NRIF signals cell death from the nucleus (R-MMU-205043). All enrichment results were displayed in Supplementary Table S5.

**Figure 6:**
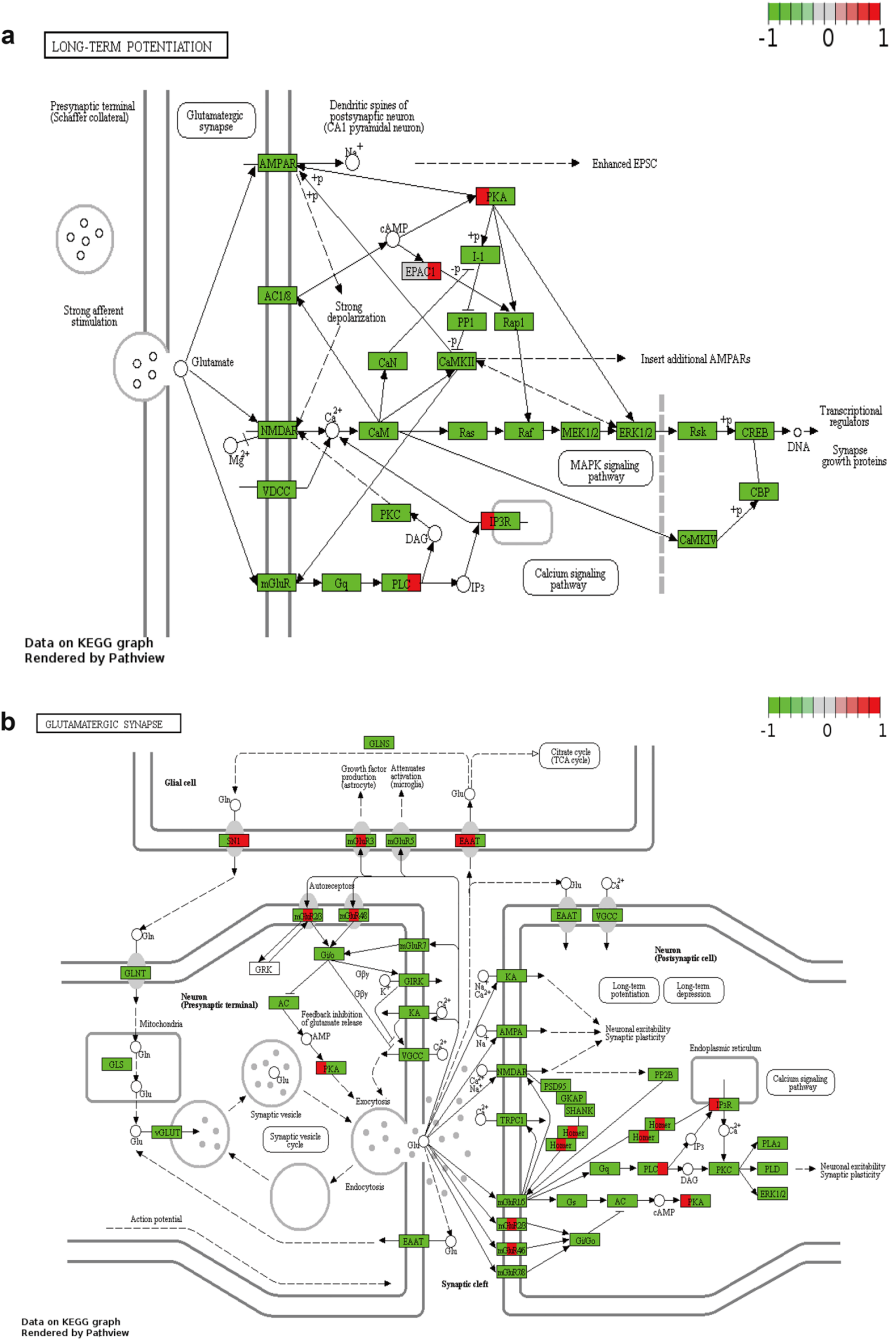
Two KEGGs related to hippocampal neurophysiology and pathology and DEGs were indicated in quotes from the KEGG database (www.kegg.jp/feedback/copyright.html.). (**a**) Long-Term Potential (mmu04720); (**b**) Glutamatergic Synapse (mmu04724). Green represents the downregulation of the gene, while red represents the upregulation of the gene.

Two KEGGs related to hippocampal neurophysiology and pathology, with DEGs indicated, are shown in Fig. 6, including Long Term Potential (mmu04720) (Fig. 6a), and Glutamatergic Synapse (mmu04724) (Fig. 6b).

### Construction of ncRNA-miRNA-mRNA ceRNA network

Competitive endogenous RNA (ceRNA) regulatory networks play a crucial role in various human diseases. We constructed a circRNA-miRNA-mRNA and a lncRNA-miRNA-mRNA triple regulatory network based on the above differentially expressed ncRNAs. The functional parts and potential mechanisms of this network were assessed by functional enrichment analysis.

**Figure 7:**
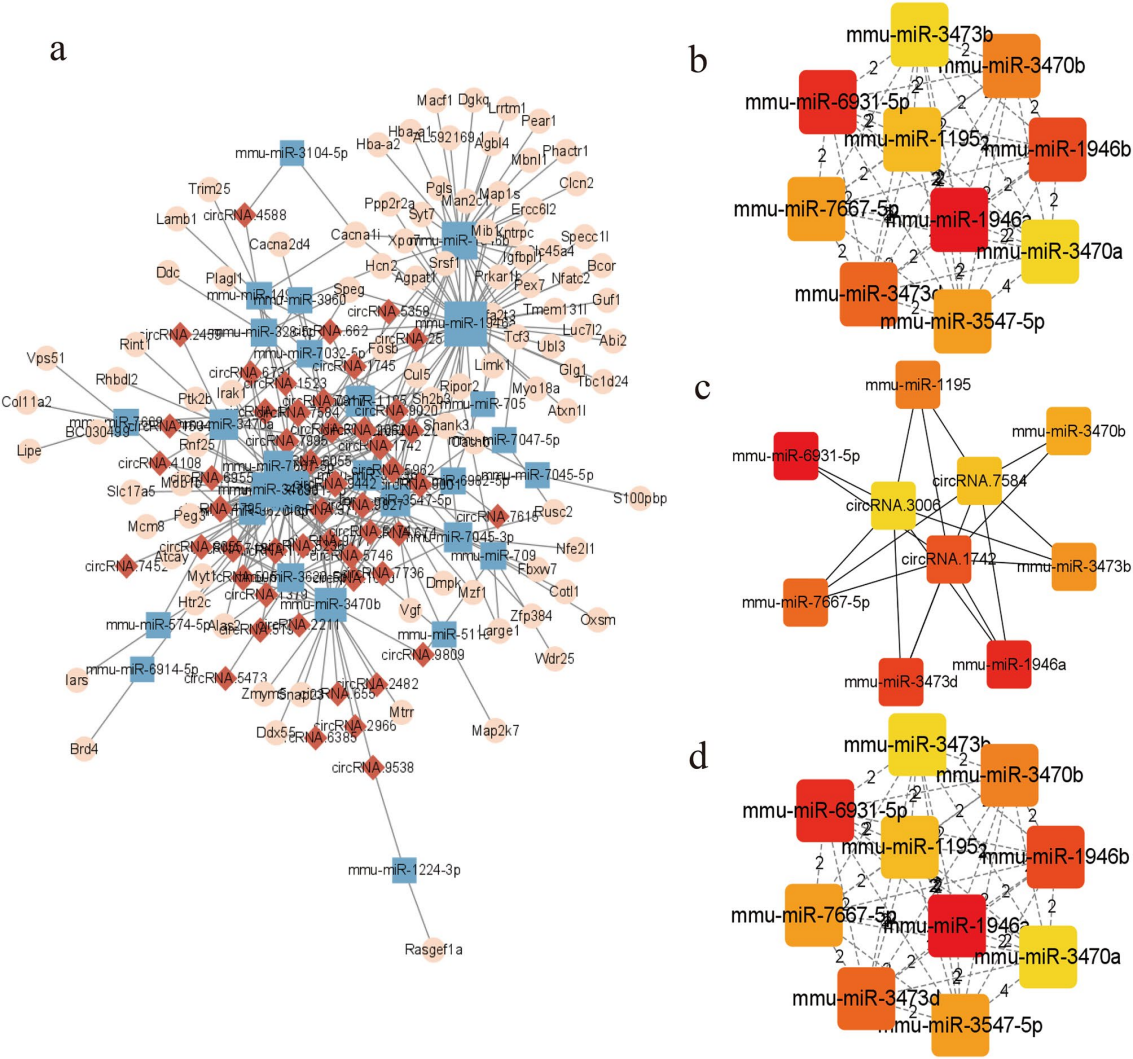
The construction of circRNA-miRNA-mRNA ceRNA networks displayed by Cytoscape (v3.9.1). (**a**) CircRNA-associated ceRNA networks. Red diamond nodes represent significantly differential circRNAs, blue rectangle nodes represent their predicted miRNAs, and pink ellipse nodes represent the predicted mRNAs. (**b**) The top 10 hub genes in the ceRNA network were calculated by the Degree method. (**c**) The top 10 hub genes in the ceRNA network were calculated by the EPC method. (**d**) The top 10 hub genes in the ceRNA network were calculated by the MCC method.

In this study, we obtained 75 differential circRNAs, 1216 miRNAs, and 333 mRNAs in the circRNA-related ceRNA regulatory network, as shown in Fig. 7a. Five circRNAs, including circRNA.1742, circRNA.3006, circRNA.4795, circRNA.7584 circRNA.9442 were considered as potential central circRNA, and 11 miRNAs, including mmu-miR-149-3p, mmu-miR-1946a, mmu-miR-1946b, mmu-miR-328-5p, mmu-miR-3470a, mmu-miR-3470b, mmu-miR-3473b, mmu-miR-3473d, mmu-miR-3547-5p, mmu-miR-6931-5p, and mmu-miR-7045-3p, were considered as key miRNAs (Fig. 7b–d). We derived related ceRNA gene pairs, such as circRNA 7584/mmu-miR-1946a/Cacna1i, circRNA 7817/mmu-miR-6931-5p/Fosb, etc. Specific information on circRNA-related ceRNA networks was provided in Supplementary Table S6, and detailed information on 12 algorithms was listed in Supplementary Table S7.

**Figure 8:**
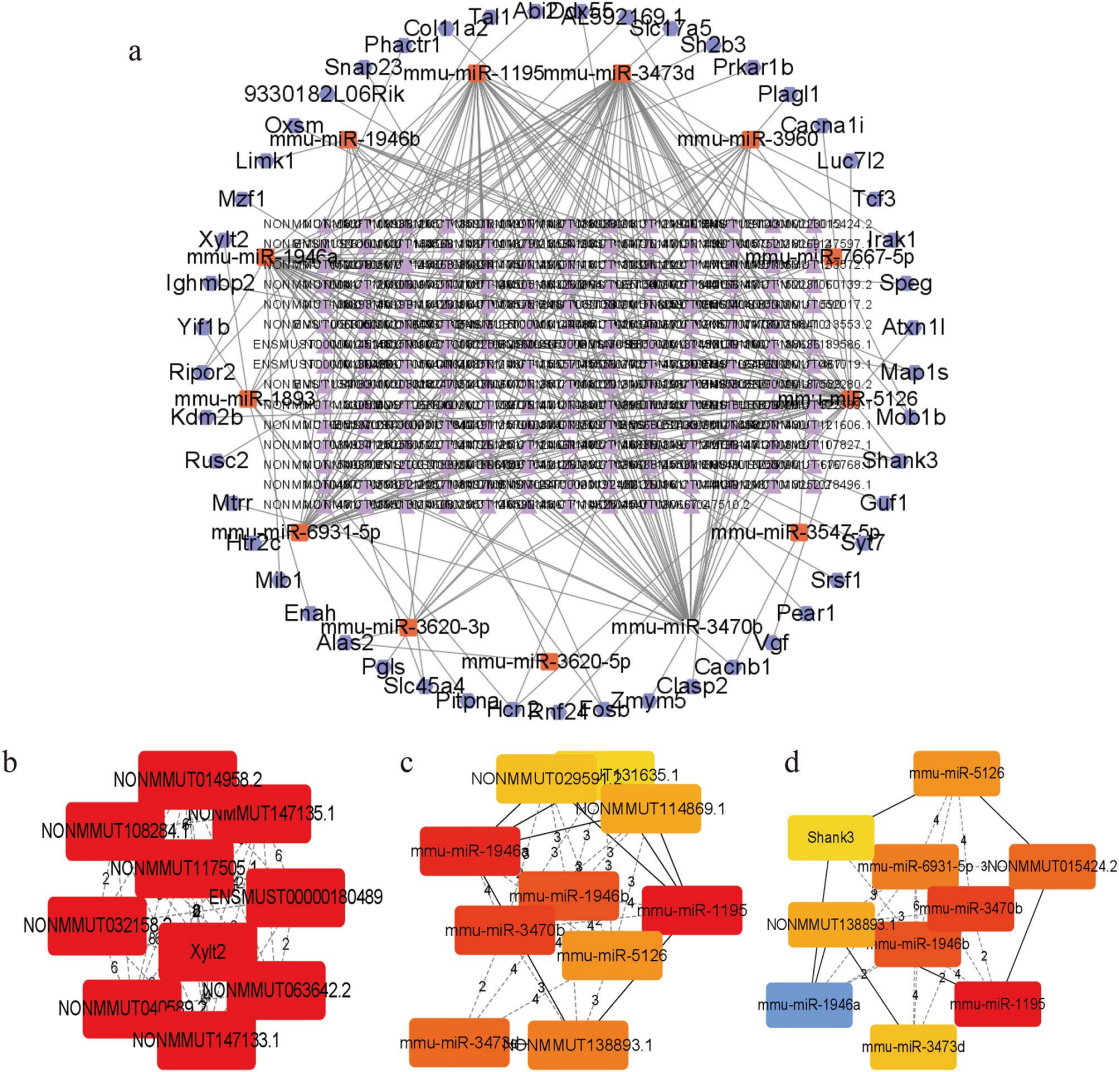
The construction of lncRNA-miRNA-mRNA ceRNA networks displayed by Cytoscape (v3.9.1). (**a**) LncRNA-associated ceRNA networks. Purple triangle nodes represent significantly differential lncRNAs, orange round rectangle nodes represent their predicted miRNAs, and dark grey hexagon nodes represent the predicted mRNAs. (**b**) The top 10 hub genes in the ceRNA network were calculated by the Clustering Coefficient method. (**c**) The top 10 hub genes in the ceRNA network were calculated by the EPC method. (**d**) The top 10 hub genes in the ceRNA network were calculated by the Stress method.

A lncRNA-associated ceRNA network containing 2337 lncRNAs,113 miRNAs, and 330 mRNAs, and the network map containing the top 500 genes are shown in Fig. 8a. After calculation, ENSMUST00000145250, ENSMUST00000180800, NONMMUT015424.2 was considered as the central lncRNA, mmu-miR-1195, mmu-miR-1893, mmu-miR-1946a, mmu-miR-3470b, mmu-miR-3473d, mmu-miR-3620-3p, mmu-miR-3960, mmu-miR-5126, mmu-miR-6931-5p were considered as hub miRNA (Fig. 8b–d). We identified several ceRNA gene pairs that may have critical roles, such as NONMMUT115700.1/mmu-miR-3473d/Mob1b, NONMMUT015424.2/mmu-miR-5126/AL592169.1, ENSMUST00000180800/mmu-miR-5126/Shank3, etc. Specific information on lncRNA-related ceRNA networks was provided in Supplementary Table S8, and detailed information on 12 algorithms was listed in Supplementary Table S9.

### Functional enrichment analysis of ceRNA networks

**Figure 9:**
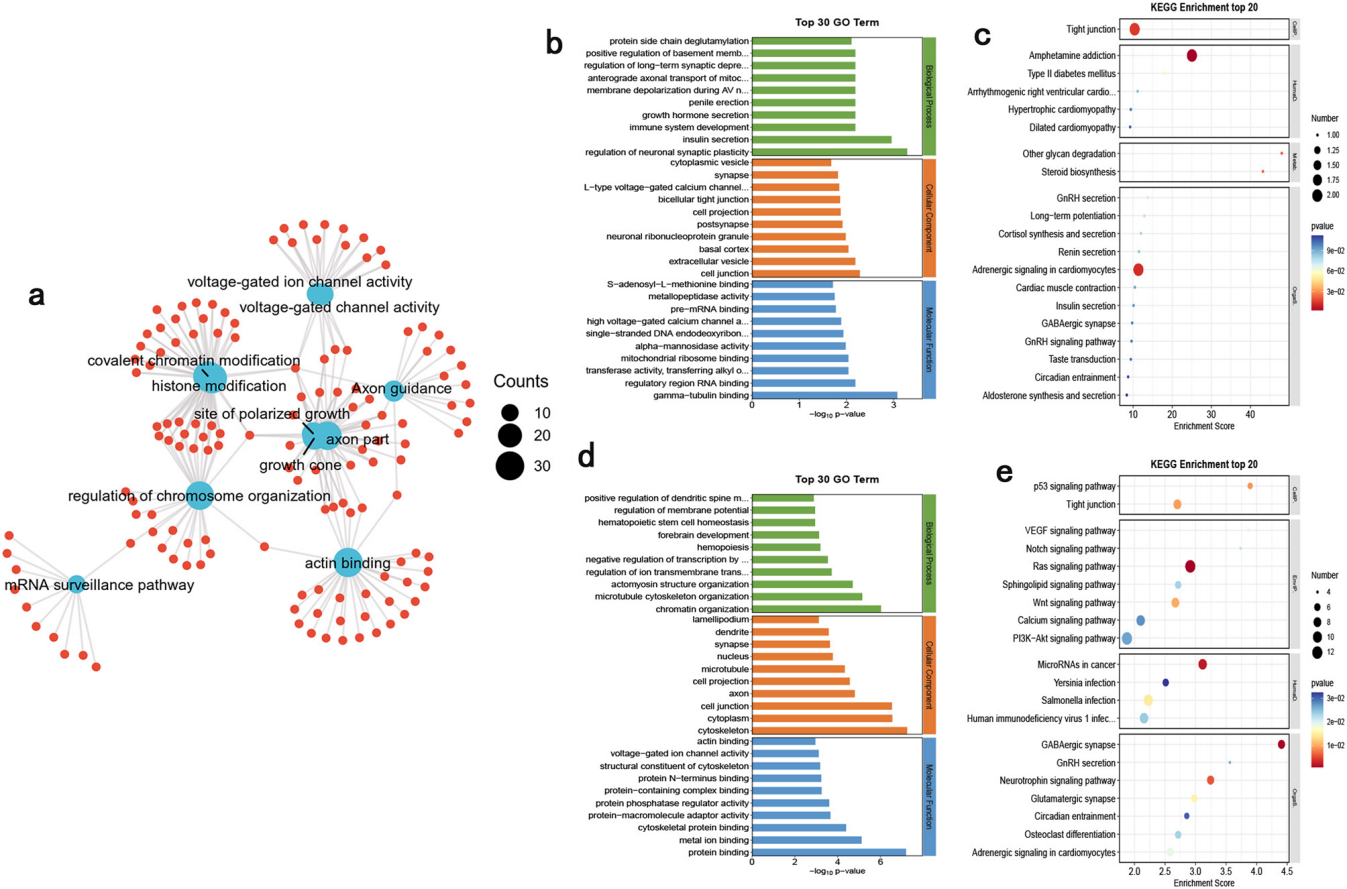
Enrichment networks analysis of mRNAs targeted circRNAs analyzed using OECloud tools (https://cloud.oebiotech.com). (**a**) The functional enrichment network of circRNA-associated ceRNA targeting mRNAs. (**b**,**c**)The number of genes in GO/KEGG term of down-regulated mRNAs targeted circRNAs. (**d**,**e**) The number of genes in GO/KEGG term of up-regulated mRNAs targeted circRNAs in the hippocampus of tx-J mice.

We performed GO/KEGG enrichment analysis by separately targeting mRNAs in two ceRNA networks. The functional enrichment network of circRNA-associated ceRNA targeting mRNAs is shown in Fig. 9a. The GO and KEGG results of down-regulated mRNA functional enrichment were demonstrated in Fig. 9b,c. GO analyses (Fig. 9b) enriched in cell junction (GO: 0030054), cell projection (GO: 0042995), synapse (GO: 0045202), cytoplasmic vesicle (GO: 0031410), and KEGG analyses (Fig. 9c) enriched in Amphetamine addiction (mmu05031), Adrenergic signaling in cardiomyocytes (mmu04261), Tight junction (mmu04530)etc. The GO and KEGG results of up-regulated mRNA functional enrichment were demonstrated in Fig. 9d,e. GO analyses (Fig. 9d) enriched in cytoplasm (GO: 0005737), protein binding (GO: 0005515), nucleus (GO: 0005634), metal ion binding (GO: 0046872), and KEGG analyses (Fig. 9e) enriched in Ras signaling pathway (mmu04014), PI3K-Akt signaling pathway (mmu04151), Salmonella infection (mmu05132), etc. The enrichment results were displayed in Supplementary Table S10.

**Figure 10:**
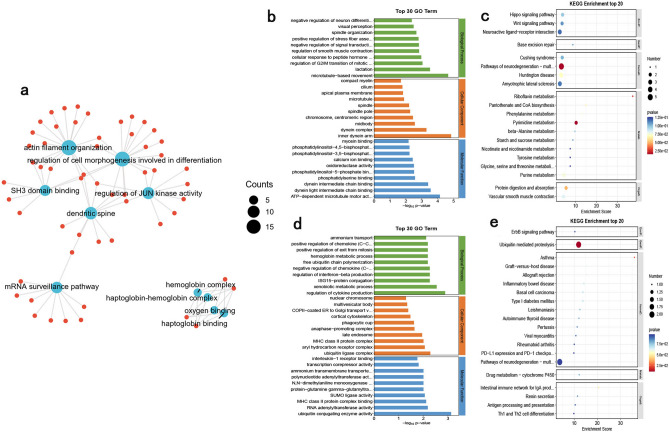
Enrichment networks analysis of mRNAs targeted lncRNAs analyzed using OECloud tools (https://cloud.oebiotech.com). (**a**) The functional enrichment network of lncRNA-associated ceRNA targeting mRNAs. (**b**,**c**) The number of genes in GO/KEGG term of down-regulated mRNAs targeted lncRNAs. (**d**,**e**) The number of genes in GO/KEGG term of up-regulated mRNAs targeted lncRNAs in the hippocampus of tx-J mice.

The functional enrichment network of lncRNA-associated ceRNA targeting mRNAs is shown in Fig. 10a. GO/KEGG results of down-regulated mRNA functional enrichment were demonstrated in Fig. 10b,c. GO analyses (Fig. 10b) enriched in oxidoreductase activity (GO: 0016491), calcium ion binding (GO: 0005509), microtubule-based movement (GO: 0007018), microtubule (GO: 0005874), apical plasma membrane (GO: 0016324), and KEGG analyses (Fig. 10c) enriched in Pathways of neurodegeneration-multiple diseases (mmu05022), Huntington disease (mmu05016), Neuroactive ligand-receptor interaction (mmu05014), etc. The GO and KEGG results of up-regulated mRNA functional enrichment were demonstrated in Fig. 10d,e. GO analyses (Fig. 10d) enriched in regulation of cytokine production (GO: 0001817), xenobiotic metabolic process (GO: 0006805), ubiquitin ligase complex (GO: 0000151), and KEGG analyses (Fig. 10e) enriched in Ubiquitin mediated proteolysis (mmu04120), Pathways of neurodegeneration-multiple diseases (mmu05022), etc. All enrichment results were displayed in Supplementary Table S11.

### Construction of ncRNA-mRNA co-expression network

**Figure 11:**
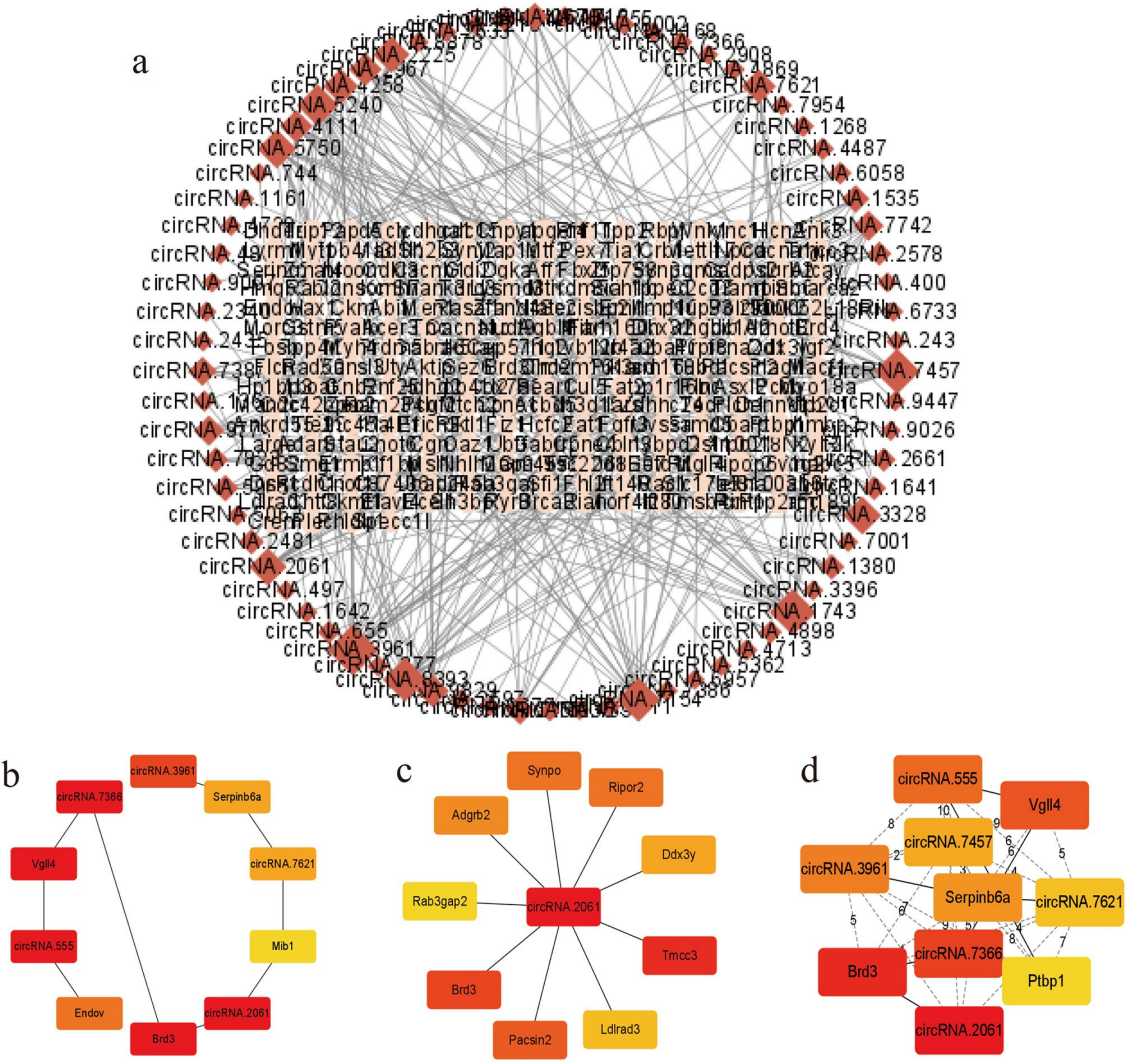
Visualizations of circRNA-mRNA co-expression network and sub-networks displayed by Cytoscape (v3.9.1). (**a**) CircRNA-mRNA co-expression network. Red diamond nodes represent circRNAS. Pink eclipse nodes represent mRNAs. (**b**) The top 10 hub genes in the circRNA-mRNA co-expression network were calculated by the MCC method. (**c**) The top 10 hub genes in the circRNA-mRNA co-expression network were calculated by the Radiality method. (**d**) The top 10 hub genes in the circRNA-mRNA co-expression network were calculated by the Stress method.

Based on the Pearson correlation coefficient |*r*|> 0.7 and the *P* < 0.05, we obtained 2051 circRNA-mRNA co-expressed gene pairs, including 92 circRNAs and 397 mRNAs, as shown in Fig. 11a. Among them, the five circRNAs with the highest correlation include “circRNA.1641”, “circRNA.2061”, “circRNA.4258”, “circRNA.4898”, “circRNA.7366”, “circRNA.7621”. The algorithm-based subnetworks are shown in Fig. 11b–d. The information on the circRNA-mRNA pairs were displayed in Supplementary Table S12, and detailed information on 12 algorithms was listed in Supplementary Table S13.

**Figure 12:**
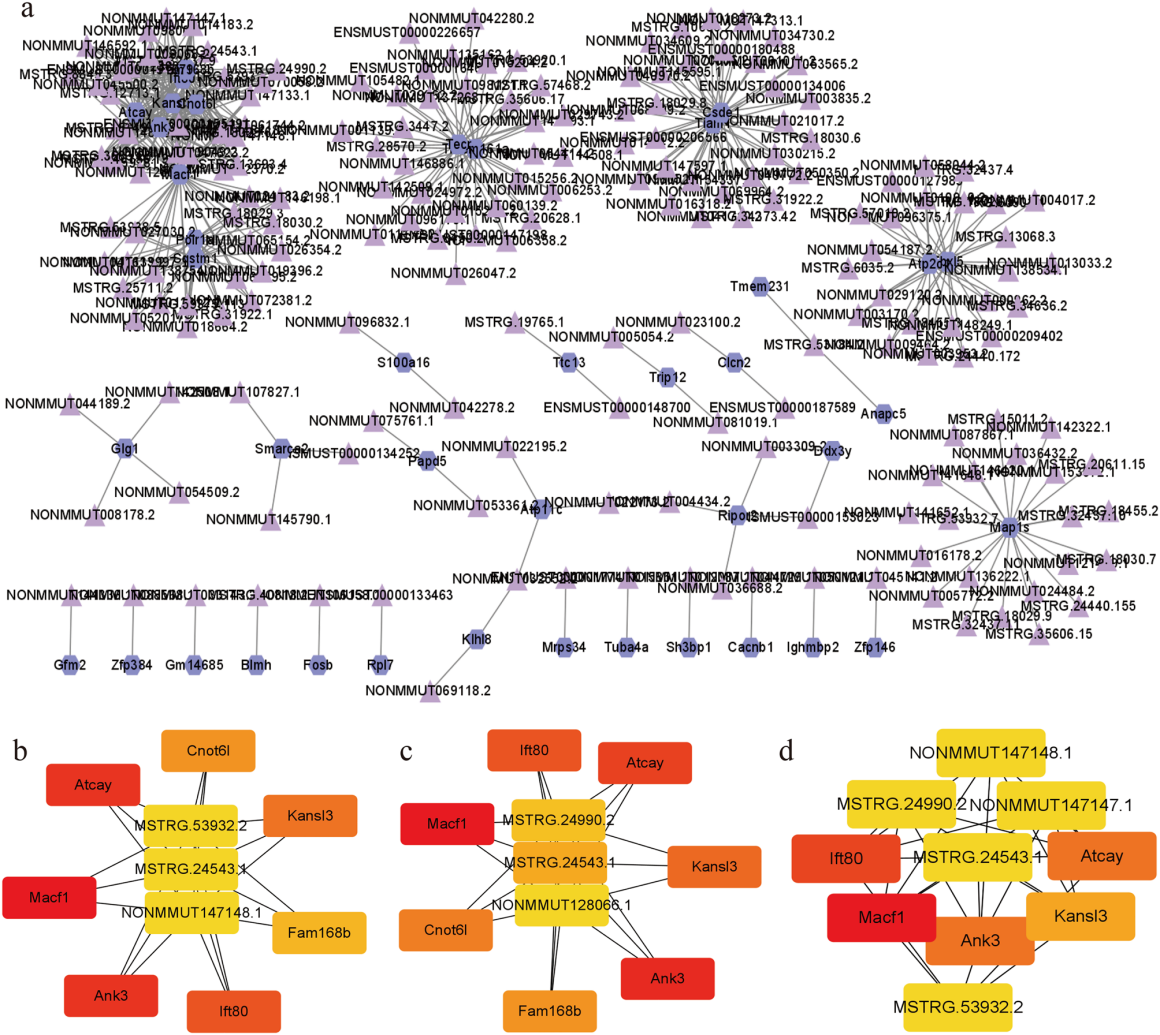
Visualizations of lncRNA-mRNA co-expression network and sub-networks displayed by Cytoscape (v3.9.1). (**a**) LncRNA-mRNA co-expression network. Purple triangle nodes represent lncRNA. Dark grey hexagon nodes represent mRNAs. (**b**) The Closeness method calculates the expression maps of the top 10 hub genes in the lncRNA-mRNA co-expression network. (**c**) The EPC method calculates the expression maps of the top 10 central genes in the lncRNA-mRNA co-expression network. (**d**) The Stress method calculates the expression maps of the top 10 hub genes in the circRNA-mRNA co-expression network.

We constructed a lncRNA-mRNA co-expression network at the same threshold with 2627 lncRNAs and 361 mRNAs in Fig. 12a. We identified eight lncRNAs as crucial lncRNAs, including “MSTRG.18029.6”, “MSTRG.24543.1”, “NONMMUT014183.2”, “NONMMUT059281.2”, “NONMMUT072370.2”, “NONMMUT072383.2”, “NONMMUT147148.1”, “NONMMUT148168.1”. The subnetworks based on the three algorithms are shown in Fig. 12b–d. The information on the lncRNA-mRNA gene pair was listed in Supplementary Table S14, and detailed information on 12 algorithms was listed in Supplementary Table S15.

## Discussion

The current work is the first transcriptome analysis of circRNAs, lncRNAs, and mRNAs in the hippocampus of the tx-J mouse model of WD. In these models, differentially expressed circRNAs, lncRNAs and mRNAs are associated with the mechanisms by which cognitive impairment occurs in WD. We focused on the participating components of non-coding RNAs in the ceRNA network and their effects on cellular functions. We analyze the characteristic involvement of non-coding RNAs in ceRNA networks regulating cognitive impairment in WD, which contributes to exploring biomarkers for the diagnosis or prognosis of cognitive impairment in WD at the molecular level.

A growing body of evidence suggests that RNA transcription and metabolism changes are critical to developing complex symptoms and diseases. Non-coding RNAs represent the majority of the organism's transcriptome, which is abundant in the central nervous system and intensely involved in the transcriptional regulation of RNAs, thus being associated with the mechanisms of many neurological symptoms and diseases^17^. Several studies have shown that coding and non-coding RNAs regulate transcription through binding miRNAs and thus affect downstream target gene expression, which is known as a competitive endogenous RNA mechanism^18^. This pattern of gene regulation can be used to explain the complexity of species or phenotypic^19^.

Studies have shown that up to 60% of WD patients have neurological or psychiatric symptoms at presentation^20^, including motor, cognitive, and behavioral deficits, and that persistent or progressive cognitive dysfunction is present, even in patients treated with "copper detoxification" and patients with or without brain copper accumulation^21^. As mentioned previously, tx-J mice are currently an ideal model for studying the transcriptome sequencing of WD and related phenotypes. In this study, we confirmed the presence of cognitive impairment in tx-J mice through a water maze test, including decreased spatial learning and memory abilities. Meanwhile, regarding morphological examination of hippocampal structures, tx-J model mice showed destruction of neuronal cells in the dentate gyrus region of the hippocampus. They significantly decreased the number of new neuronal cells. It indicates that there is detectable cognitive impairment in tx-J mice compared with controls. On the one hand, this evidence of cognitive impairment can improve animal models' scientificity and strengthen sequencing results' reliability.

In this study, we performed the differential expression analysis of mRNA\circRNA\lncRNA, constructed a PPI expression network, and the ncRNA-related ceRNA network and ncRNA/mRNA co-expression network in the hippocampal tissue of WD model tx-J mice for the first time. We screened 361 DEGs. Many of these significantly differentially expressed target genes are closely associated with cognitive impairment pathology. We found that some mRNAs that miRNA targets in ncRNA-associated ceRNA regulatory networks were also differentially expressed mRNAs, such as Fosb, Shank3, and Cacna1i, etc.

Fosb is an activity-dependent transcription factor that accumulates and is retained during chronic cellular activity due to its long half-life^22^. Fosb plays a critical role in regulating hippocampal memory; Studies have confirmed that the Fosb family regulates many addiction-related targets of crucial target genes through histone modifications^23^. It has also been demonstrated that over-expression of Fosb in the hippocampus may affect learning and memory^24^. A study examining Fosb regulation of gene expression in AD model mice with cognitive dysfunction suggests that Fosb may suppress the expression of c-Fos, an early gene critical for plasticity and cognition, by binding to promoters and triggering histone deacetylation and that the long half-life of Fosb makes it a possible cause of the persistence of cognitive deficits^25^. As an excitatory postsynaptic scaffolding protein, SHANK3 acts on various postsynaptic density proteins to regulate postsynaptic neurotransmitter receptors and signaling molecules^26^. A study based on the prediction of single-gene variant levels in Autism Spectrum Disorder (ASD) showed that SHANK3 mRNA levels affect synaptic transmission in the hippocampus of mice, leading to possible long-term depression and long-term impairment of learning and memory^27^. It was found that the voltage-gated calcium (Cav) channel gene CACNA1I (Cav3.3) is considered a risk factor for schizophrenia and that variants in this gene lead to disruption of neuronal excitability and brain network activity, affecting processes such as transmitter release, sensation, memory, and sleep^28^.

Non-coding RNA is a class of RNA molecules that do not encode proteins and account for approximately 98–99% of the total RNA in mammalian genomes^29^. There is increasing evidence that non-coding RNAs play important regulatory roles by actively interacting with other molecules; for example, a non-coding RNA interacts with one or more target molecules to regulate cells or pathways to affect normal physiological or pathological processes, including the regulation of neurological diseases^30^. CircRNAs are a class of covalently closed, single-stranded cyclic molecules without 5'-caps and 3'-polyadenylated tails^31^, which are stable, abundant, and conserved^32^ and have unique potential for medical research. In this study, we identified the circRNAs profile in hippocampus tissue with WD, and 99 significant DECs were screened out. In particular, mmu_circ_0001859 and mmu_circ_0000242 were strongly related to Axon guidance, Wnt signaling pathway, Calcium signaling pathway, MAPK signaling pathway, Focal adhesion, Neurotrophin signaling pathway, TGF-beta signaling pathway, Cell cycle, ErbB signaling pathway, Notch signaling pathway, p53 signaling pathway, Fc gamma R-mediated phagocytosis, etc.

Studies have confirmed the biological functions of circRNAs, such as participating in transcriptional regulation in the nucleus, acting as endogenous adsorbers of miRNAs, templates for protein or peptide translation, and regulators of gene expression^33^. CircRNA is exceptionally abundant in the mammalian brain, which is upregulated at the overall level during neuronal differentiation, highly enriched in neurosynapses, and plays a vital role in the development of neuropsychiatric disorders^34^. A study based on deep RNA sequence analysis systematically elucidated the cranial circRNA-associated ceRNA mechanism in an AD model mouse (APP/PS1 mice) and found that the circRNA-associated ceRNA network in this model mouse is mainly involved in dendritic development and memory (Sorbs2) and mouse neurodevelopment (ALS2), which provides new ideas for the clinical diagnosis and treatment of AD^35^. The first exploration of hippocampal circRNA expression profiles in aged mice with POCD suggests that mmu_circRNA_22058 and circRNA_44122\Egfr ceRNA network or circRNA_22673\Prkacb ceRNA network may have meaningful involvement in the course of POCD disease^36^. In this study, we identified 99 significantly differentially expressed circRNAs in hippocampal tissue of WD model tx-J mice compared to controls. We constructed a ceRNA network based on differentially expressed circRNAs and performed functional enrichment analysis. We performed GO and KEGG analyses on both up-and down-regulated genes of circRNA-associated ceRNA networks respectively, and identified a series of enriched terms associated with neurological diseases and cognitive processes. In our results, GO of up-regulated genes was enriched in the cytoplasm (GO: 0005737), protein binding (GO: 0005515), nucleus (GO: 0005634), and metal ion binding (GO: 0046872). In contrast, KEGG was enriched in the Ras signaling pathway (mmu04014), PI3K-Akt signaling pathway (mmu04151), Calcium signaling pathway (mmu04020), etc. GO of down-regulated genes were enriched in cell junction (GO: 0030054), cell projection (GO: 0042995), synapse (GO: 0045202), and cytoplasmic vesicle (GO: 0031410), and KEGG was enriched in Amphetamine addiction (mmu05031), Adrenergic signaling in cardiomyocytes (mmu04261), Tight junction (mmu04530), Circadian entrainment (mmu04713). We identified several ceRNA networks that may contribute to the progression of cognitive impairment in WD. For example, in the ceRNA network with mmu-miR-6931-5p as the core and Fosb as the target gene, the upstream 44 circRNAs may bind mmu-miR-6931-5p as a sponge to regulate the expression of Fosb target genes.

Unlike circRNAs, LncRNAs are langer than 200 nucleotides in length that have 5’ end caps and 3’ end multimeric tails so that they are easily recognized by oligonucleotide-based RNA sequencing^37^. The expression of lncRNAs in the brain is tissue-specific^38^, and studies have confirmed the presence of regional and cell-specific expression patterns of lncRNAs in cognitively relevant memory brain regions such as the hippocampus, prefrontal cortex, and amygdala^39^, such as lncRNA Gm9968, which is significantly enriched in mouse hippocampus tissue, plays an important role in diseases such as Alzheimer's disease and epilepsy. Many lncRNAs with cognitive relevance in vivo or in vitro models have been demonstrated. For example, LncRNA Rian can reduce LIMK1 expression by negatively regulating miR143-3p. Thus modulation of the LncRNA Rian/miR143-3p/LIMK1 axis can improve cognitive dysfunction after sevoflurane anesthesia^40^. A study on the mechanism of LncRNA MEG3 action on cognitive function in AD model rats showed that upregulation of LncRNA MEG3 inhibited neuronal damage, reduced Aβ positive expression, and improved inflammatory indexes in AD rats, improving cognitive function as well^41^. In mouse models of vascular cognitive impairment, LncRNA TUG1 can bind and interact with BDNF proteins, and its overexpression can lead to cognitive dysfunction in mice after VCI^42^. We identified 2627 DELs in the hippocampal tissue of WD model tx-J mice and performed functional enrichment analysis in the constructed lncRNA-associated ceRNAs. Among them, the upregulated target genes GO were enriched in the regulation of cytokine production (GO: 0001817), xenobiotic metabolic process (GO: 0006805), ubiquitin ligase complex (GO: 0000151), late endosome (GO: 0005770), ubiquitin-conjugating enzyme activity (GO: 0061631), transcription corepressor activity (GO: 0003714), KEGG enrichment in Ubiquitin mediated proteolysis (mmu04120), Pathways of neurodegeneration—multiple diseases (mmu05022). Down-regulated expression of target genes GO is enriched in oxidoreductase activity (GO: 0016491), calcium ion binding (GO: 0005509), microtubule-based movement (GO: 0007018), microtubule (GO: 0005874), apical plasma membrane (GO: 0016324), cilium (GO: 0005929). KEGG analysis is enriched in Pathways of neurodegeneration—multiple diseases (mmu05022), Huntington's disease (mmu05016), Amyotrophic lateral sclerosis (mmu05014), Neuroactive ligand-receptor interaction (mmu04080). For example, in the ceRNA network targeting SHANK3, the upper lncRNA NONMMUT015424.2 (lncRNA D130020L05Rik) may use mmu-miR-5126 as a sponge to regulate the transcriptional expression of SHANK3 by binding to it. Our predicted ncRNA-ceRNA network model may contribute to the development of cognitive impairment in WD, and the function of these networks requires further experimental validation.

The study presented in this paper has several limitations. Firstly, the small sample size may limit the generalizability of the findings. Additionally, results obtained from animal models may not be entirely representative of the transcriptional profile of all phenotypes of Wilson's disease (WD), including female mice and human patients. Secondly, the assessment of spatial learning and memory in mice may not have been comprehensive enough. Furthermore, this study solely investigated the transcriptional profile of the hippocampus in the WD animal model, and other copper deposition tissues were not examined, which means that it was not possible to investigate gene expression in different tissues. Moreover, the study's results lack experimental validation, including genes with significant expression differences and predictive network models, which requires robust experiments to provide evidence for the results. Although our study sheds light on the mechanisms of cognitive impairment in WD based on transcriptomics, we acknowledge that the expression regulation mechanism of transcription molecules is complex and requires further research, such as the regulation of epigenetic modification.

In conclusion, investigating the pathological phenotypic mechanisms of diseases is a challenging and time-consuming undertaking. However, the advancement of high-throughput sequencing technology has significantly facilitated the exploration of molecular mechanisms underlying diseases. Our examination of the protein–protein interaction (PPI) network and non-coding RNA competing endogenous RNA (ncRNA-ceRNA) network in the hippocampal tissue of tx-J mice, a model of Wilson's disease (WD), carries important implications for identifying potential biomarkers and target values essential for diagnosing, treating, and developing drugs for cognitive impairment in WD.

## Methods

### Animals

The study utilized ten male tx-J mice (C3HeB/FeJ-Atp7b tx-J/J) weighing 20 ± 2 g and ten male wild-type mice (C3HeB/FeJ) aged 8–10 weeks, acquired from the Jackson Laboratory through Beijing Vital River Laboratory Animal Technology, Ltd. All mice were individually housed in cages with controlled humidity (50–70%), room temperature (18–22 °C), and separate air supplies, with ad libitum access to food and water under an alternating 12 h light/dark cycle for eight weeks. For this study, there was an overlap of animal subgroups; three of the four tx-J mice underwent high-throughput sequencing after MWM testing, four of the remaining seven mice underwent hippocampal histopathology testing, and the remaining three underwent qRT-PCR experiments. The wild-type mice were also treated according to this protocol. Estrogen has been shown to influence neuroplasticity in several brain regions, regulate and mediate neuronal synapses and hippocampal formation, and affect learning and memory^43^. Thus, male mice were chosen as research subjects to minimize these effects.

The methods used in this study were performed in accordance with the relevant guidelines and regulations of the ARRIVE2.0 guidelines^44^. The Experimental Animal Ethics Committee of Anhui University of Traditional Chinese Medicine reviewed and approved the animal use protocol (Permit Number: AHUCM-mouse-2021125).

### Morris water maze (MWM) test

The Morris water maze experiment is routinely used to assess spatial learning and memory abilities in rodents, which consisted of a black circular pool (50 cm high and 90 cm in diameter) filled with 45 cm high water at 21–23 °C and mixed with a white non-toxic, odorless dye, in which a black circular table fixed 1 cm underwater in the center of the fourth quadrant. Four mice from each of the two groups were selected for the experiment. In the positioning navigation experiment, the two groups of mice were put into the water from the 45°angle position facing the pool wall in any of the four east, west, south, and north quadrants and tested four times a day. If the mice climbed up to the hidden platform within the 60 s and stayed on the platform for more than 5 s, they were considered to have found the platform. The time from water entry to platform finding was recorded as the escape latency. If the mice did not find the platform within the 60 s, they were guided to the platform and stayed there for 10 s, and the escape latency was recorded as 60 s. Compare the escape latency of the opposite platform quadrant, the average escape latency of three quadrants except for the platform quadrant, the swimming distance of the opposite platform quadrant, and the total swimming distance except for the platform quadrant on the fifth day between the two groups.

### Hippocampus histopathology

Four mice from each group were selected for hippocampus histopathology. Mice were anesthetized by intraperitoneal injection of sodium pentobarbital (2 mL/kg; Shanghai Chemical Reagent Company) after fasting for 12 h and taken bilateral hippocampus tissues, fixed with 4% paraformaldehyde, dehydrated in ethanol and xylene, embedded in clear paraffin, sectioned, delayed, stained with hematoxylin–eosin, further dehydrated and sealed, and observed under a light microscope.

### RNA extraction and high-throughput RNA sequencing

After isolating hippocampus tissue in the WD and control groups on ice boxes, we performed deep-sequencing of ribosome RNA samples from the hippocampus of three control and three tx-J mice.

Total RNA of Peripheral Blood Mononuclear Cells (PBMC) was prepared using MiRNeasy Mini Kit (Qiagen, Germany). Afterward, the RNA Clean XP Kit (Cat#A63987, Beckman Coulter, USA) and RNase-Free DNase Set (Cat#79254, Qiagen, Germany) were used to purify the total RNA. The purified RNA was quantified using two instruments for detecting RNA content and quality: NanoDrop 2000 (Thermo Fisher Scientific, USA) and an Agilent Bioanalyzer 2100 (Agilent Technologies, USA). According to the manufacturer's instructions, the libraries were prepared using the truseq® chain total RNA sample preparation kit (Illumina, USA). Qubit 2.0 fluorometer was used to quantify the library. Furthermore, these libraries were verified by Agilent 2100 Bioanalyzer. After confirming the size and molar concentration of the inserted fragment, the libraries were diluted to 10 pm with CBOT to generate clusters and sequenced on Illumina HiSeq 2500 (Illumina, USA). The library was constructed and sequenced in OG Biotech Inc (Aoji Biotech, Shanghai, China).

### Identification and qualification of the expression level of RNAs

By using FastQC (v. 0.11.3, http://www.bioinformatics.babraham.ac.uk/projects/fastqc)^45^, the RNA-Seq reads were quantified. Seqtk (https://github.com/lh3/seqtk) removes the adapter sequence of Illumina TruSeq, mm10 ribosomal RNA reads, and low-quality reads. Then, using Hisat2 (version: 2.0.4), trimmed reads were mapped to the mouse reference genome (mm10) downloaded from Ensembl. The count of each gene in aligned reads was detected by StringTie (version 1.3.0). With Perl script, normalization of gene counts and fragments per kilobase of transcript per million mapped reads (FPKM) was performed.

For circRNAs, All valid sequencing data were processed using the following steps: 1) The data were aligned to the mouse genome (http://genome.ucsc.edu/)^46^ by BWA-MEM software (version: 2.0.4); 2) The reads that were contiguously aligned to the genomes were discarded; 3) The unmapped reads were analyzed explicitly for back-splice junction sites using CIRI algorithms to identify the possible circRNAs^47^ and counts were normalized by SRPBM (Spliced Reads Per Billion Mapping). Genes generating individual circRNAs were identified by matching the genomic locations of circRNAs to those detected by TopHat/Cufflinks using BED tools^48^. The conservation of circRNA between humans and mice was analyzed using the UCSC Liftover tool. It was utilized to map the 3´ and 5´ flank coordinates of each dysregulated circRNAs to the human genome coordinates. The criterion for homology was detecting the splice sites within ± 2 nucleotide distance around the putative human sites^49^.

For the identification of lncRNAs, single lane paired reads of 2 × 150 bp (PE150) in length were assembled using the transcript assembly software StringTie to remove known mRNAs and transcripts < 200 bp in length. The prediction software: Coding Potential Calculator (https://github.com/biocoder/cpc) and Coding Non-Coding Index (https://github.com /www-bioinfo-org/CNCI#install-CNCI) for lncRNA prediction of the remaining transcripts were then filtered from the lncRNA dataset. The expression levels of lncRNAs were measured by fragments per kilobase of exon model per million mapped reads (FPKM), and the expression abundance of known genes in different samples was calculated from FPKM values.

### Differential expression analysis of RNAs

EdgeR method was used to determine the differentially expressed genes, *P* < 0.05 and |log2FC|> 1.5. The Venn diagram is plotted on Jvenn (http://jvenn.toulouse.inra.fr/app/example.html)^50^. The volcano map was performed using the OECloud tools at https://cloud.oebiotech.com. The heat map was done on Heatmapper (http://www.heatmapper.ca/)^51^.

### Real-time quantitative PCR (qRT-PCR) validation of circRNA candidates

Real-time qRT-PCR was performed in the following reaction system: 3.6 μL RNase-free H_2_O, 0.2μL forward primer (5 μM), 0.2 μL reverse primer (5 μM), 1 μL cDNA template, and 5 μL SYBR green supermix (Abclonal, Wuhan, China) at 95 °C for the 30 s, followed by 15 s reaction, and then reacted at − 60 °C for 15 s and 70 °C for 15 s, cycling 40 times. Primers were designed and synthesized by OG Biotech Inc (Aoji Biotech, Shanghai, China). The relative expression levels of the circRNAs were depicted as 2^−∆∆CT^.

### PPI network construction and analysis

The STRING database (https://cn.string-db.org/) is an integrated database that affords information on interactions of protein–protein, not only including experimental interactions but also predicted interactions, which was used to predict the interaction of DE mRNAs with proteins. Cytoscape (v3.9.1) is used for constructing the PPI network. Using the MCAO plugin with the standard sets, the modules of the PPI network were identified with Cytoscape. GO, and KEGG pathway enrichment analysis was performed using the OECloud tools (https://cloud.oebiotech.cn). Visualize DEGs and related paths through the Pathview website (https://pathview.uncc.edu/).

### Construction and analysis of ceRNA

All sequences of mice-derived miRNAs were obtained from miRBase version 21^52^. OG-Biotech's custom-built software, which is based on miRanda software and RNAhybrid, was adopted to predict miRNA targets that can interact with the DECs and DELs.

The number of predicted binding miRNAs was counted for all circRNAs. The mRNA target for predicted binding miRNAs was collected from miRdana^53^, targetScan^54^, and mirTarbase^55^ database. The Venny was employed to obtain a strongly-supported mRNA target predicted by at least two databases. DECs and mRNAs that share the same miRNA binding site represented circRNA-miRNA-mRNA interactions.

For the cis-acting role of lncRNAs acting on neighboring target genes, coding genes 10 kb/100 kb upstream and downstream of lncRNA were searched for, and their functions analyzed. For the trans-acting role of lncRNA, identified based on expression level, the expressed correlation between lncRNAs and coding genes was calculated with the R function "cor. test". The ceRNA network was visually displayed by Cytoscape software.

The hub genes are calculated and displayed using the relevant algorithms in the Cytohubba plugin^56^. These 12 algorithms include Degree, Edge Percolated Component (EPC), Maximum Neighborhood Component (MNC), Density of Maximum Neighborhood Component (DMNC), Maximum Clique Centrality (MCC), and six centralities (Bottleneck, EcCentricity, Closeness, Radiality, Betweenness, and Stress) based on shortest paths and Clustering Coefficient. All algorithms and resulting network diagrams are presented in the Supplementary Figs. S1 and S2.

### Gene ontology (GO) enrichment and KEGG pathway analysis

For GO analysis, the biological process (BP), cellular component (CC), and molecular function (MF) analyses were performed. To further understand the function of the DE circ-/lnc-/mRNAs, the Gene Ontology (GO) and Kyoto Encyclopedia of Genes and Genomes (KEGG) pathways enriched were analyzed using OECloud tools (https://cloud.oebiotech.com) to reveal the high-level function and utility of the transcriptome of tx-J mice, the such as cells, organisms, and ecosystems. Significant enrichment terms for GO and pathways were picked out according to the threshold of *P* < 0.05.

### Construction and analysis of circRNA-/lncRNA-/mRNA co-expression network

Based on the matrix data of DELs/DECs/DEGs, the Pearson correlation coefficients between DEL/mRNAs and DEC/mRNAs were calculated, respectively, and the threshold value of |r|> 0.7 and *P* < 0.05 to obtain an up-/down-regulated co-expression list between DEL/mRNAs and DEC/mRNAs. The co-expression network visualization was calculated and displayed using the 12 algorithms in the cytohubba plugin. All algorithms and resulting network diagrams are presented in the Supplementary Figs. S3 and S4.

### Statistical analysis

Statistical analyses were performed using Graphpad Prism 8 (Chicago, USA). The results are presented as the mean ± standard deviation (SD). The student's t-test was performed on the remaining data, and *P* < 0.05 was considered statistically significant.

## Supplementary Information

Supplementary Figure 1.

Supplementary Figure 2.

Supplementary Figure 3.

Supplementary Figure 4.

Supplementary Table 1.

Supplementary Table 2.

Supplementary Table 3.

Supplementary Table 4.

Supplementary Table 5.

Supplementary Table 6.

Supplementary Table 7.

Supplementary Table 8.

Supplementary Table 9.

Supplementary Table 10.

Supplementary Table 11.

Supplementary Table 12.

Supplementary Table 13.

Supplementary Table 14.

Supplementary Table 15.

Supplementary Legends.

## Data Availability

The raw sequence data reported in this paper have been deposited in the Genome Sequence Archive (Genomics, Proteomics & Bioinformatics 2021) in National Genomics Data Center (Nucleic Acids Res 2022), China National Center for Bioinformation / Beijing Institute of Genomics, Chinese Academy of Sciences^57,58^. The sequences in the following format: https://ngdc.cncb.ac.cn/gsa/browse/CRA010567 and the working link to the bioproject as https://ngdc.cncb.ac.cn/bioproject/browse/PRJCA012480.
