# Supplementary figures and images for: Comprehensive analysis of the coding and non-coding RNA transcriptome expression profiles of hippocampus tissue in tx-J animal model of Wilson's disease
Source: Sci Rep. 2023 Jun 7;13:9252. doi: 10.1038/s41598-023-36503-8 (PMC10247767; doi:10.1038/s41598-023-36503-8)

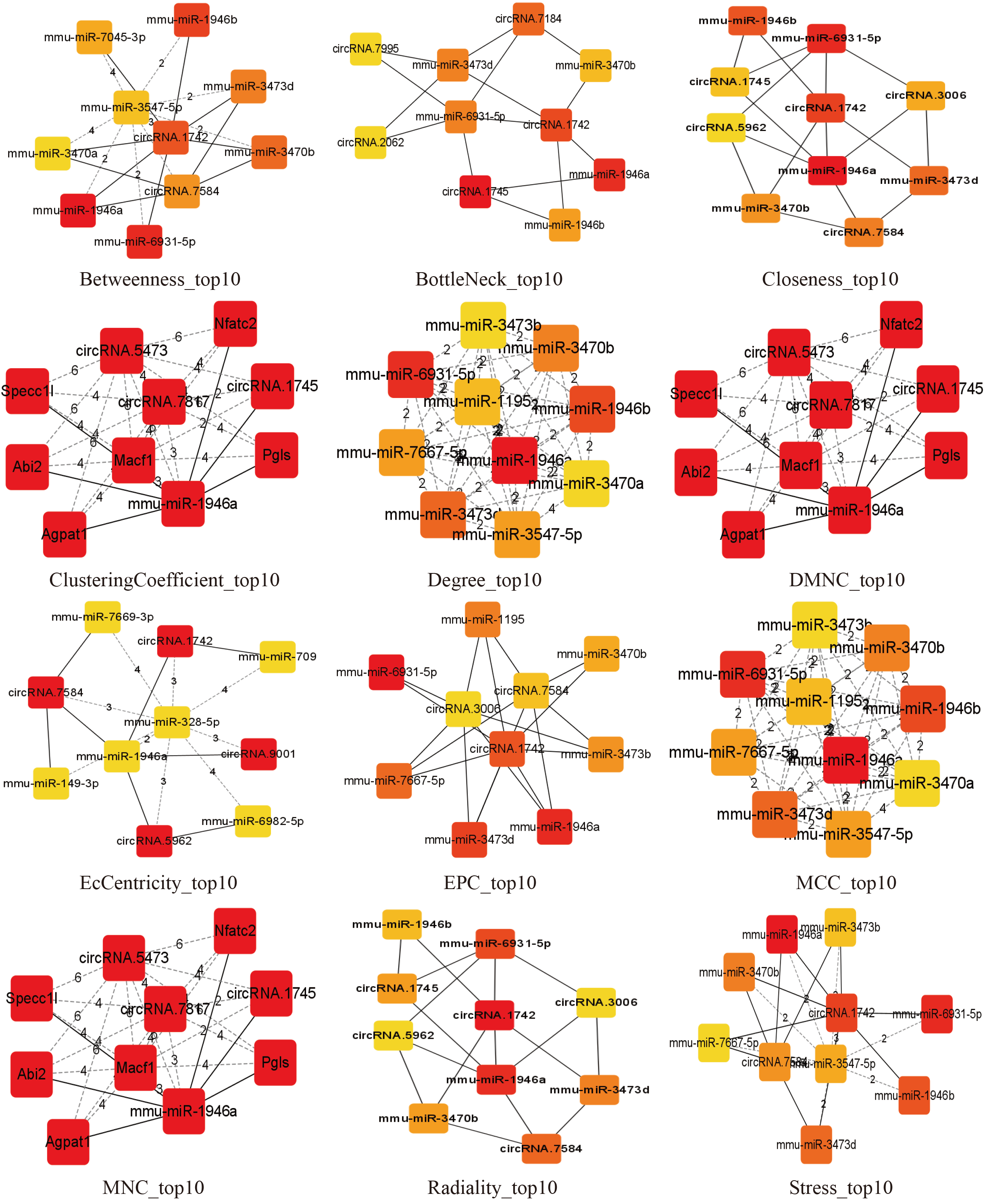

Supplement: Supplementary file 1 — Supplementary Figure 1. [file 41598_2023_36503_MOESM1_ESM.tif]

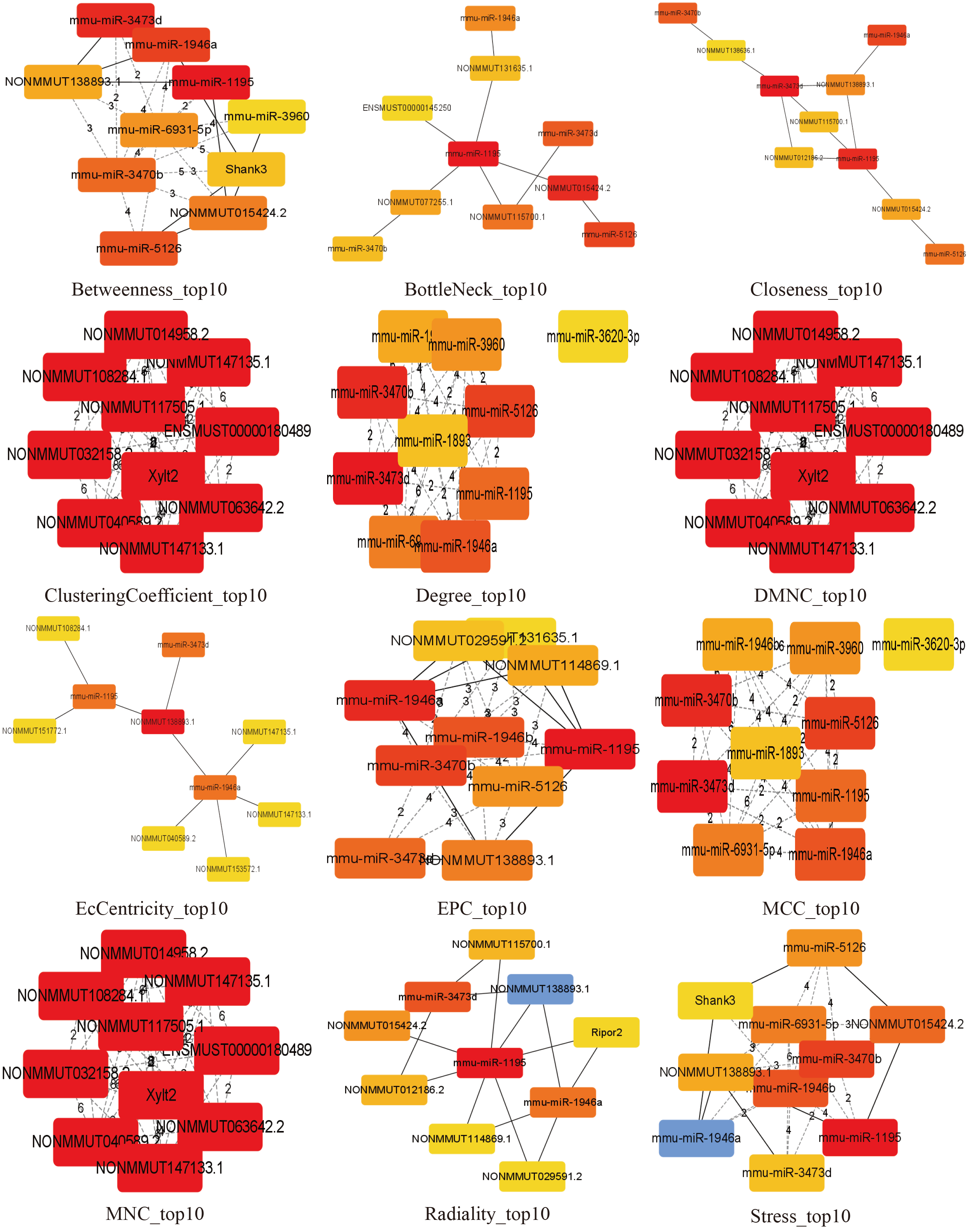

Supplement: Supplementary file 2 — Supplementary Figure 2. [file 41598_2023_36503_MOESM2_ESM.tif]

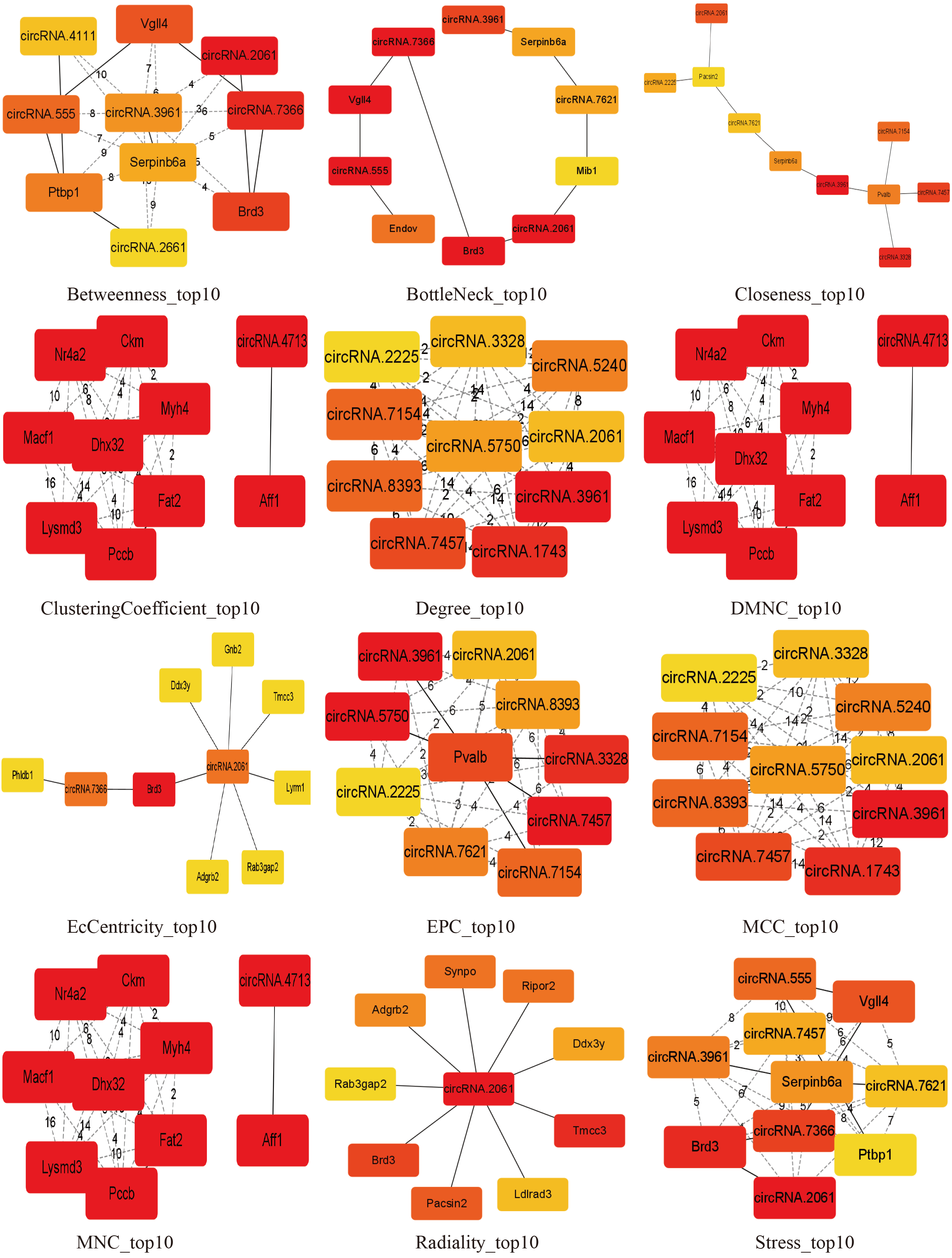

Supplement: Supplementary file 3 — Supplementary Figure 3. [file 41598_2023_36503_MOESM3_ESM.tif]

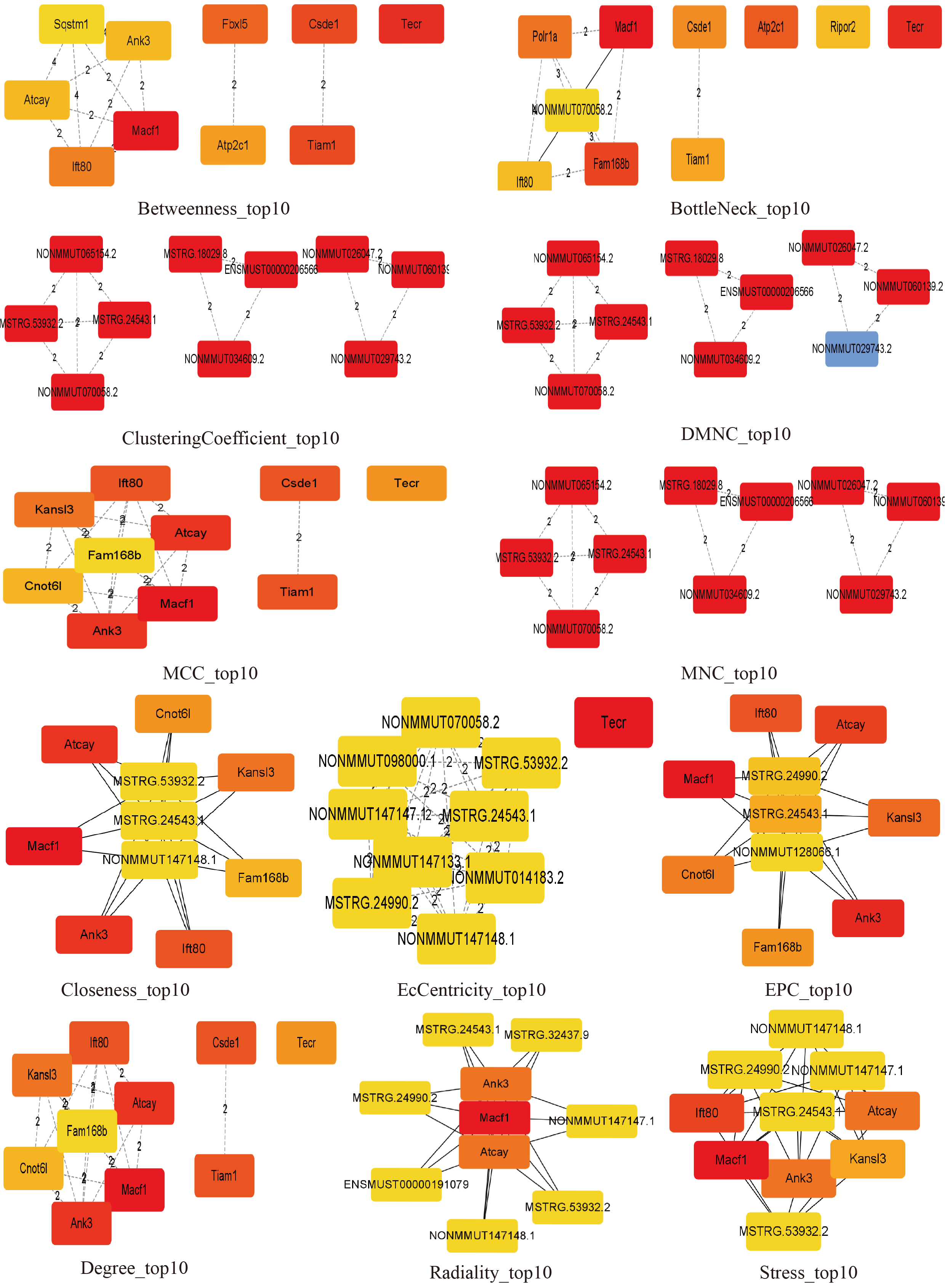

Supplement: Supplementary file 4 — Supplementary Figure 4. [file 41598_2023_36503_MOESM4_ESM.tif]
