# Supplementary material for: Comprehensive analysis of the coding and non-coding RNA transcriptome expression profiles of hippocampus tissue in tx-J animal model of Wilson's disease
Source: Sci Rep. 2023 Jun 7;13:9252. doi: 10.1038/s41598-023-36503-8 (PMC10247767; doi:10.1038/s41598-023-36503-8)
Supplement: Supplementary file 20 — Supplementary Legends. [file 41598_2023_36503_MOESM20_ESM.docx]

**Supplementary Figure 1:** The 12 algorithms and resulting network diagrams of circRNA-miRNA-mRNA ceRNA networks.

**Supplementary Figure 2:** The 12 algorithms and resulting network diagrams of lncRNA-miRNA-mRNA ceRNA networks.

**Supplementary Figure 3:** The 12 algorithms and resulting network diagrams of circRNA-mRNA co-expression networks.

**Supplementary Figure 4:** The 12 algorithms and resulting network diagrams of lncRNA-mRNA co-expression networks.

**Supplementary Table 1:** Detailed information on DEGs.

**Supplementary Table 2:** Detailed information on DELs.

**Supplementary Table 3:** Detailed information on DECs.

**Supplementary Table 4:** Detailed information on interactions of the PPI network.

**Supplementary Table 5:** All enrichment results of DEGs.

**Supplementary Table 6:** Detailed information on interactions of the circRNA-miRNA-mRNA ceRNA network.

**Supplementary Table 7:** Detailed information on 12 algorithms of the circRNA-miRNA-mRNA ceRNA network.

**Supplementary Table 8:** Detailed information on interactions of the lncRNA-miRNA-mRNA ceRNA network.

**Supplementary Table 9:** Detailed information on 12 algorithms of the lncRNA-miRNA-mRNA ceRNA network.

**Supplementary Table 10:** Detailed information on GO/KEGG analysis of the circRNA-associated ceRNA network.

**Supplementary Table 11:** Detailed information on GO/KEGG analysis of the lncRNA-associated ceRNA network.

**Supplementary Table 12:** Detailed information on interactions of the circRNA-mRNA co-expression network.

**Supplementary Table 13:** Detailed information on 12 algorithms of the circRNA-mRNA co-expression network.

**Supplementary Table 14:** Detailed information on interactions of the lncRNA -mRNA co-expression network.

**Supplementary Table 15:** Detailed information on 12 algorithms of the lncRNA-mRNA co-expression network.
